# Environmental and Anthropogenic Factors Shape Major Bacterial Community Types Across the Complex Mountain Landscape of Switzerland

**DOI:** 10.3389/fmicb.2021.581430

**Published:** 2021-03-11

**Authors:** Johanna Mayerhofer, Daniel Wächter, Pierluigi Calanca, Lukas Kohli, Tobias Roth, Reto Giulio Meuli, Franco Widmer

**Affiliations:** ^1^Molecular Ecology, Agroscope, Zurich, Switzerland; ^2^Swiss Soil Monitoring Network, Agroscope, Zurich, Switzerland; ^3^Climate and Agriculture, Agroscope, Zurich, Switzerland; ^4^Hintermann & Weber AG, Reinach, Switzerland

**Keywords:** amplicon sequencing, bacterial barcode, biogeography, cluster analysis, bipartite network, land-use, environmental selection

## Abstract

Mountain areas harbor large climatic and geographic gradients and form numerous habitats that promote high overall biodiversity. Compared to macroorganisms, knowledge about drivers of biodiversity and distribution of soil bacteria in mountain regions is still scarce but a prerequisite for conservation of bacterial functions in soils. An important question is, whether soil bacterial communities with similar structures share environmental preferences. Using metabarcoding of the 16S rRNA gene marker, we assessed soil bacterial communities at 255 sites of a regular grid covering the mountainous landscape of Switzerland, which is characterized by close location of biogeographic regions that harbor different land-use types. Distribution of bacterial communities was mainly shaped by environmental selection, as revealed by 47.9% variance explained by environmental factors, with pH (29%) being most important. Little additional variance was explained by biogeographic regions (2.8%) and land-use types (3.3%). Cluster analysis of bacterial community structures revealed six bacterial community types (BCTs), which were associated to several biogeographic regions and land-use types but overall differed mainly in their preference for soil pH. BCT I and II occurred at neutral pH, showed distinct preferences for biogeographic regions mainly differing in elevation and nutrient availability. BCT III and IV differed only in their preferred soil pH. BCT VI occurred in most acidic soils (pH 3.6) and almost exclusively at forest sites. BCT V occurred in soils with a mean pH of 4 and differed from BCT VI in preference for lower values of organic C, total nitrogen and their ratio. Indicator species and bipartite network analyses revealed 3,998 OTUs associating to different levels of environmental factors and BCTs. Taxonomic classification revealed opposing associations of taxa deriving from the same phyla. The results revealed that pH, land-use type, biogeographic region, and nutrient availability were the main factors shaping bacterial communities across Switzerland. Indicator species and bipartite network analyses revealed environmental preferences of bacterial taxa. Combining information of environmental factors and BCTs yielded increased resolution of the factors shaping soil bacterial communities and provided an improved biodiversity framework. OTUs exclusively associated to BCTs provide a novel resource to identify unassessed environmental drivers.

## Introduction

Topography of mountain areas creates diverse ecosystems due to variation of several factors such as temperature gradients as well as patchiness of nutrient availability, moisture, and geology. This results in a large number of habitats harboring a high biological diversity. For instance, 20% of all higher plant species in Europe occur in the alpine zones, which encompass only 3% of the total area of Europe (Väre et al., 2003). However, in comparison to plant diversity little is known about the biodiversity of bacteria in soils of European alpine areas. Correlations of plant and soil bacterial diversity have been reported (e.g., Prober et al., 2015) and suggest that alpine habitats may also harbor a high bacterial diversity.

Biogeographic distribution patterns, such as maximum species richness at mid-elevation, have been found for different macroorganisms including plants and small mammals (Grytnes and McCain, 2013), however, only few and inconsistent results have been reported for microorganisms. Some studies have addressed the effect of environmental factors on soil bacterial communities of mountainous regions, covering large ranges of elevation (Fierer et al., 2011; Singh et al., 2014; Wang et al., 2015; Yashiro et al., 2016; Terrat et al., 2017; Karimi et al., 2018). In France, drivers of prokaryotic alpha diversity and distribution of phyla were assessed in a regular sampling grid across the entire country. It covered part of the Western Alps and included a difference in elevation of approximately 2,500 m (Terrat et al., 2017; Karimi et al., 2018). The most important factors, which correlated with the distribution of bacterial phyla have been in descending order pH, land-use, soil texture, soil nutrients, and climate (Karimi et al., 2018). In the same system, bacterial richness was most strongly influenced by physico-chemical properties, such as pH, clay content and the C/N ratio (Terrat et al., 2017). In a study of bacterial community structures covering an elevation range of 2,200 m within 700 km^2^ in the western Swiss Alps the factor explaining most of the variance in bacterial community structures was by far soil pH (Yashiro et al., 2016). Additional important factors were, the hydrogen index, which was defined as the proportion of hydrocarbons versus total organic carbon and represents the labile fraction of soil organic matter, and the number of days below freezing during the growing season (Yashiro et al., 2016). Soil pH was also the most important factor explaining soil bacterial community structures in soils located between 3,106 and 4,479 m.a.s.l. on Mt. Shegyla on the Tibetan Plateau and furthermore they correlated with clay content, total nitrogen and soil organic carbon (Wang et al., 2015). On Mt. Halla in South Korea, bacterial community structures correlated most strongly with elevation, which correlated with mean annual temperature and precipitation, across a difference in elevation ranging from 100 to 1,950 m.a.s.l. In this study the soil pH range has been relatively narrow and acidic (3.67 to 4.95), which may explain the limited influence of pH on bacterial communities (Singh et al., 2014). In contrast, no significant effect of elevation has been observed across an elevation gradient in eastern Peruvian Andes, which was chosen because of its small pH range of 3.4 to 4.4, similar plant cover and moisture availability (Fierer et al., 2011). In summary, in the above-mentioned studies of mountainous regions, pH has been identified as the main driver of bacterial communities unless narrow pH gradients were selected. Furthermore, effects of soil nutrients, soil texture, and climate differed among the studied areas.

These findings have suggest an important role of environmental selection processes that lead to biogeographic distribution patterns of soil bacterial communities. Biogeographic patterns may result from stochastic factors, environmental filtering, and dispersal (Hanson et al., 2012). A well-studied biogeographic distribution pattern of macro- as well as microorganisms has been referred to as the distance-decay relationship, which assumes that with increasing geographic distance communities become more dissimilar (Hanson et al., 2012). Distance-decay relationships have been found for bacterial communities at a regional scale, for instance across France (Chemidlin Prévost-Bouré et al., 2014), in the state of New York (Barnett et al., 2019) and in Northern China (Feng et al., 2019). However, it is unclear whether the distance-decay relationship is also important for the biogeographic distribution of soil bacterial communities across the complex alpine landscape of Switzerland, which encompasses a relatively small but highly structured area of 41,285 km^2^.

The alpine landscape of Switzerland represents an ideal area for studying the driving factors of soil bacterial community structures in mountainous regions. It is characterized by large gradients in soil, climatic, and geographic factors and includes a heterogeneous geography with the mountain ranges of the Alps and the Jura, their valleys as well as the lowlands (Central Plateau) between them. In addition, a variety of different land-use types, such as, forests, meadows, alpine meadows, arable land, and settlements are established across the country. It has been shown that bacterial community structures differ among land-use types in different regions, for instance, across Europe (Szoboszlay et al., 2017) or in Malaysia (Tripathi et al., 2012). However, the number and kind of land-use types may differ among regions making generalizations of the effect of land-use on bacterial communities difficult. In many cases, environmental factors were identified as the stronger drivers of soil bacterial community structures in comparison to land-use type (Lauber et al., 2008; Tripathi et al., 2012). Nevertheless, the assessment of effects of land-use types on bacterial communities under different management intensities may reveal anthropogenic impacts on bacterial communities, which is an important issue regarding biodiversity conservation and preservation of soil quality (Joimel et al., 2016). It remains unclear if and to which extent bacterial community structures differ among land-use types across Switzerland in an area with diverse environmental gradients also within land-use types, such as forests that occur across a large elevational range.

To study bacterial communities in Swiss soils we took advantage of the established sampling system of the Swiss biodiversity monitoring (BDM), which has been designed for macroorganisms and aims at assessing the dynamics of macrobial biodiversity across the country. The goal of the BDM is to establish a baseline of biodiversity, on which policy targets for conservation strategies may be defined and controlled (Weber et al., 2004). A variety of different organisms, such as vascular plants, bryophytes, mollusks, butterflies, and breeding birds, are monitored across different sampling grids (Hintermann et al., 2000). For the present study, we used 255 of the 1,600 BDM sites from the BDM sampling grid that aims at assessing species diversities of plants, mosses and mollusks in small areas of defined land-use types (grid Z9). The sites of the BDM are located on a regular grid, where every year one fifth of the plots is sampled. Several research questions have been addressed using this scheme, including plant species homogenization over time and the effect of nitrogen deposition on plant diversity (Bühler and Roth, 2011; Roth et al., 2015). Monitoring of soils and soil microorganisms have not yet been considered in the BDM.

In the present study, soil bacterial communities were assessed using metabarcoding of the 16S rRNA gene region covering V3 and V4. Communities were determined from replicate soil cores, which were collected at each of 255 sites, and most analyses were based on means for each site. The following research questions were pursued. (1) Which soil, geographic, and climatic factors best explain differences among bacterial communities across Switzerland? (2) Do land-use types and biogeographic regions harbor characteristic and different bacterial community structures? (3) Can clusters of similar bacterial community structures be defined as bacterial community types (BCTs) and assigned to environmental preferences? (4) Which bacterial taxa are associated to the above-mentioned factors?

## Materials and Methods

### Description of the Sites, Climatic and Geographic Factors, and Plant Data

**FIGURE 1:**
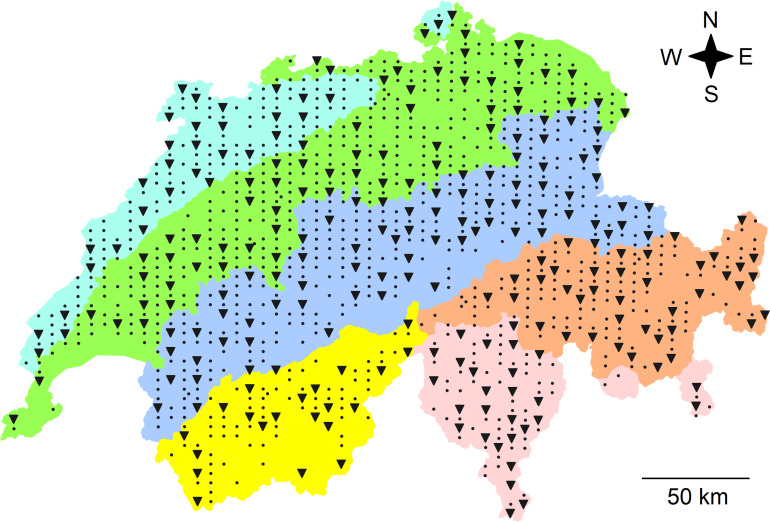
Map of Switzerland with the locations of the 255 sites, where soil bacterial communities were sampled (triangles) and of all the other 903 sites (dots) of the soil sampling campaign of the Swiss Biodiversity Monitoring (BDM). Colors represent biogeographic regions (BGR; from North to South): the Jura Mountains (light blue), the Central Plateau (green), the Northern Alps (dark blue), the Eastern (orange), and Western (yellow) Central Alps representing the central ridge of the Alps as well as the Southern Alps (rose). The entire country includes an area of 41,285 km^2^ and the extension of the sampling area was 222 km from North to South and 343 km from West to East. Data for the map were retrieved from Federal Office for the Environment (2001).

In total, 255 sites were sampled along a regular grid with distances of 4 and 6 km among latitudinal and longitudinal gridlines, across the entire country of Switzerland (41,285 km^2^) between April and October in 2014 (121 sites) and in 2015 (134 sites; Figure 1). The area of the study revealed an east-west extension of 343 km and a north-south extension of 222 km. The sites are part of the sampling grid with approximately 1,600 sites for species richness in defined habitat types of the ongoing Biodiversity Monitoring of Switzerland (Hintermann et al., 2000; Weber et al., 2004). Site-specific information, including land-use type and geographic coordinates were obtained from the BDM and recorded as previously described (Weber et al., 2004; Bühler and Roth, 2011). This data was used to infer further site-specific factors from national databases. Sites were assigned to five different land-use types including forest, meadow, alpine grassland, arable land, and settlement (Bühler and Roth, 2011). Sites that were assigned as “unused” were considered as alpine grassland because of similar environmental conditions such as elevation. Geographic coordinates were used to assign each site to a biogeographic region, i.e., Jura, Central Plateau, Northern Alps, Western Central Alps, and Eastern Central Alps as well as the Southern Alps (Federal Office for the Environment, 2001). Twenty-three different environmental factors, which included geographic, climatic, plant-derived and soil properties (see paragraph below), were considered (Table 1). Geographic data including elevation, slope, and exposition were obtained from the Federal Statistical Office (BFS) and were based on the GEOSTAT and RIMINI terrain models in 2007. Average and variability (long-term monthly mean and inter-annual standard deviation of monthly values) of climatic factors, i.e., temperature, precipitation and solar radiation were based on gridded data spanning the years 1981 and 2010, which were retrieved from MeteoSwiss (Federal Office of Meteorology and Climatology MeteoSwiss, 2018). Monthly values were aggregated to obtain yearly mean values. The gridded fields (2 × 2 km horizontal resolution) were generated using a variety of statistical methods (Dürr and Zelenka, 2009; Frei, 2014; Isotta et al., 2014). Indicator values based on recorded vascular plant species were calculated for soil pH (R), soil nutrients (N), soil moisture (F), and variability of soil moisture (W; Landolt, 2010). These indicator values are based on data which show that the distribution of plants is related to environmental conditions and that the plant community composition provides a measure for these conditions. Indicator values were developed for Central Europe (Ellenberg et al., 1992) and have been adapted for the Swiss flora including indicator values for about 5,500 plant species (Landolt, 2010). To determine the indicator values for each site, mean indicator values of all recorded plant species were calculated for each site. Environmental factors were available for 194 to 255 of the sites and a complete set of environmental factors was available for a reduced set of 160 sites (Table 1 and Supplementary Figure 1). This reduced set also represented the Swiss area and included a similar percentage of different land-use types and biogeographic regions as the full set of 255 sites (Supplementary Table 1).

**TABLE 1:** Descriptive statistics of environmental factors including means, stand deviations (SD), medians, and minimum and maximum values as well as the number of sites for which data were available (n).

Environmental factor	*n*	Mean	*SD*	Median	Min	Max
Organic carbon [%]^a^	225	5.6	5.2	3.9	0.1	35.9
Total nitrogen [%]^a^	231	0.4	0.3	0.3	0.02	2.6
Carbon-nitrogen ratio^a^	225	13.6	5.8	12.5	3.2	58
pH^a^	236	5.6	1.3	5.6	2.9	7.8
Bulk density [g dried fine^b^ soil/cm^3^]^a^	238	0.7	0.3	0.7	0.1	1.4
Clay [%]^a^	194	20.6	10.6	18.4	0.5	58.2
silt [%]^a^	194	32.8	10.2	31.8	12.1	74
Sand [%]^a^	255	40.9	16.8	41.9	0	80.1
Soil water content [%]^a^	238	26.9	11.5	25.7	4.3	75.5
DNA content [ng/g dry weight of soil]^a^	238	21.5	20.8	15.5	2.3	169.6
Plant ind.^c^ for moisture	255	3.0	0.2	3.1	2.3	4
Plant ind.^c^ for variability of moisture	255	1.7	0.3	1.7	1	3
Plant ind.^c^ for pH	255	3	0.5	3.1	1.5	4.4
Plant ind.^c^ for nutrients	255	3	0.6	3	1.9	4.3
Elevation [m.a.s.l.]	255	1194.8	647.6	1074	267	2741
Slope [gradian]	255	18.2	12.9	17	0	49
Exposition [gradian]	224	204.8	119.5	189.5	3	400
Mean annual temperature [°C]^d^	255	6.1	3.3	6.7	−3	12.2
SD of inter-annual temperature [°C]^de^	255	1.8	0.1	1.8	1.3	2
Mean annual precipitation [mm/y]^df^	255	1386.2	371.3	1335.5	617.1	2565.6
SD of inter-annual precipitation [mm/mo]^dg^	255	62.9	22.1	56.2	34.9	148.2
Mean annual solar radiation [W/m^2^/y]^df^	255	1689.5	73.3	1668.4	1563.6	1899.9
SD of inter-annual solar radiation [W/m^2^/mo]^dg^	255	19.9	1.8	19.5	17.4	27.1

*SD, standard deviation.*

*^*a*^Based on means for each site.*

*^*b*^Dried and sieved soil (<2 mm).*

*^*c*^Plant indicator.*

*^*d*^Based on values from between 1981 and 2010.*

*^*e*^Mean of monthly standard deviations.*

*^*f*^Mean of yearly sums.*

*^*g*^Mean of standard deviation of monthly sums.*

### Soil Sampling and Assessment of Soil Properties

At each site, a circle with a radius of 3.5 m from the grid point was set up for soil sampling. Plant data were assessed within a circle with a radius of 1.78 m from the same center as described in the BDM (Bühler and Roth, 2011). One soil sample was obtained from each, the north, west, south and east point of the circle in an area defined by the radii 3.0 and 3.5 m. Movement on the circle to the right or the left was allowed if an obstacle prevented sampling at the specific spot. Soil cores with a diameter of 5 cm were drawn from the upper 20 cm of the soil using a Humax device (GreenGround AG, Switzerland), which includes a plastic (polyvinylchloride, 0.2 mm) cover inside the coring device. This allowed containing the soil sample within the PVC cover for protection during transportation and storage. Within 48 h after arrival in the laboratory, soil cores were cut into halves and a transect of approximately 1 cm depth along the whole sample (0–20 cm) was cut off the inner part of one half. This soil sample was sieved to 2 mm and about 0.5 g were frozen for DNA extraction (see below). The remaining soil was used for physicochemical soil analyses including pH, total organic carbon, total nitrogen, organic carbon to nitrogen ratio, bulk density of fine soil, as well as sand, silt, clay and water content. Soil was dried at 40°C for 48 h and then sieved to 2 mm to separate the fine and the coarse fraction of the soil. Bulk density was calculated as the weight of the dried fine fraction of soil (<2 mm) divided by the volume of the entire soil sample (g/cm^3^). For the determination of soil pH, 10 g of dried soil were suspended in 0.01 M CaCl_2_ with a ratio of 1:2.5. After equilibration of the suspension for 2 h, the pH was measured using a pH-meter including an Expert Pro-ISM electrode (SevenMulti, Mettler-Toledo, Switzerland). Total nitrogen and total carbon were determined using 0.5 g of finely ground soil and dry combustion with a TruSpec CN analyzer (Leco, Germany). Total inorganic carbon (calcium carbonate) content was determined in soils with pH greater than 6.3 by measuring the volume of CO_2_ production after adding an excess of hydrochloric acid (Harris et al., 2001). Organic carbon was calculated by subtracting 12% of the inorganic carbon from the total carbon for soils with a pH greater than 6.3, while for soils below 6.3 total carbon and organic carbon were assumed to be equivalent. Carbon to nitrogen ratio was calculated by dividing organic carbon by total nitrogen. Water content was gravimetrically determined over night at 105°C. Contents of clay, silt and sand were determined by sedimentation after removal of humus using H_2_O_2_ (International Organization for Standardization, 2009).

### Soil Processing for DNA Extraction and DNA Extraction

For DNA extraction 0.5 g of sieved fresh soil (≤2 mm) was transferred to a 2 ml safe-lock Eppendorf tube, mixed with 0.5 g glass beads (0.1–0.11 mm diameter, Sortarius, Tagelswangen, Switzerland), and suspended in 1.3 ml of extraction buffer [2% CTAB, 20 mM EDTA (pH 8), 2M NaCl, 100 mM Tris (pH 8), and 2% Polyvinylpyrrolidone (PVP-40)]. The fresh soils were fixed by mixing them with glass-beads and extraction-buffer and immediate storage at −20°C until DNA extraction. DNA was extracted following a modified protocol (Bürgmann et al., 2001; Hartmann et al., 2005). DNA extracts were quantified using the Quant-iT PicoGreen dsDNA Assay kit (Invitrogen, Carlsbad, CA, United States) using a Cary Eclipse fluorescence spectrophotometer (Varian, Inc., Palo Alto, CA, United States) and diluted to 5 ng/μl. Soil DNA content (dry weight equivalent) was used as a proxy for soil microbial biomass. In total, 1,020 soil samples, i.e., four replicates of each of the 255 sites, were obtained, however, 10 of the 1,020 samples were excluded due to errors during sample processing resulting in a final number of 1,010 samples.

### Metabarcoding

Amplicon sequencing of the V3–V4 region of the prokaryotic (bacterial and archaeal) 16S rRNA gene was performed to assess bacterial communities using a modified version of the primer pair 341F and 806R (Frey et al., 2016). Forward and reverse primers were tagged with adapters CS1 or CS2 of the Fluidigm Access Array System (Fluidigm, South San Francisco, CA, United States) to allow multiplexing. PCR was performed in a volume of 25 μl and included 20 ng of soil DNA, 1 × GoTaq Flexi Buffer (Promega, Madison, WI, United States), 2.5 mM MgCl_2_, 0.6 mg/ml bovine serum albumin, 0.2 mM dNTP (Promega, Madison, WI, United States), 0.2 μM of each primer and 1.25 units of the GoTaq G2 Hotstart Polymerase. PCRs were conducted in 96–well plates and each plate included one negative control, which included purified, autoclaved and UV-irradiated water instead of soil DNA extract. PCR conditions included one initial step of denaturation at 95°C for 5 min followed by 30 cycles of denaturation at 94°C for 40 s, annealing at 58°C for 40 s and elongation at 72°C for 1 min. The reaction was completed by a 10 min final elongation at 72°C. Four independent PCR reactions were performed per sample, pooled and paired-end sequenced using the Illumina MiSeq v3 platform (Illumina Inc., San Diego, CA, United States) at the Génome Québec Innovation Center at the McGill University (Montréal, Canada).

### Bioinformatic and Statistical Analyses

Quality control, OTU clustering and taxonomic classification was performed using a pipeline (Supplementary File 1) mainly based on UPARSE (Edgar, 2013) and MOTHUR (Schloss et al., 2009) similar to a previously published (Frey et al., 2016) and modified pipeline (Mayerhofer et al., 2017). In the present study, taxonomic assignment was performed using the SILVA database release 132 (Quast et al., 2013) using the command “classify.seqs” in MOTHUR. All non-bacterial sequences, i.e., sequences classified as archaea or plant organelles were removed. The majority of statistical analyses, unless specified otherwise, were performed in Rstudio v1.0.136 using R v3.3.1 (RStudio-Team, 2015; R-Core-Team, 2016). Correlations of environmental factors were assessed using Pearson correlation coefficient and based on site-means with pairwise exclusion of missing data. In order to ensure that the community analyses were not biased by differences in sequencing depth, a mantel test based correlation of Bray Curtis (BC) community dissimilarities with subsampling to the lowest number of sequences (i.e., 3,276 sequences) and with relative OTU abundances was calculated. The subsampling approach was performed with the function “dist.shared” in MOTHUR with 1,000 iterations. The Mantel test (function “mantel” in vegan) revealed a strong correlation (*r* = 0.996, *p* = 0.0001), revealing that sequencing depth did not affect subsequent community analyses (Oksanen et al., 2018). Pairwise Bray Curtis community dissimilarities (BC) were calculated for samples and sites based on relative sequence abundance of OTUs for each sample or site-means using the function “vegdist” from vegan. Within-site similarities of bacterial communities were obtained by calculating BC similarities of samples to the respective site centroid using the function “betadisper” from vegan. For some of the environmental factors such as climatic data, elevation and plant derived indicator values only one value for each site was available. Therefore, subsequent analyses were assessed using Bray Curtis dissimilarities based on mean relative sequence abundances of OTUs for each site. Differences of BC dissimilarities among land-use types and biogeographic regions and the respective pairwise tests were assessed using overall and pairwise PERMANOVA in the function “adonis” from vegan and the function “pairwise.perm.manova” from RVAideMemoire (Hervé, 2018). A possible impact of the unequal number of sites per group on the PERMANOVA was tested using subsampling procedure to the smallest group sizes with 10,000 iterations. Because very similar results were obtained with the subsampling only the original PERMANOVA were included in the manuscript. Constrained community analyses were performed using canonical analysis of principal coordinates based on discriminant analysis (CAP) with the function “CAPdiscrim” and the maximum number of principal coordinate axes considered (“mmax”) set to 200 in BiodiversityR (Kindt and Coe, 2005). A linear distance-based model was constructed using the function “DISTLM” in Primer7 in a stepwise manner with the Akaike information selection criterion (AICc) (Clarke and Gorley, 2015), and correlations of BC dissimilarity with each environmental factor separately was also obtained from the same analysis. The separate addition of each of the factors “land-use type” and “biogeographic region” to the AICc selected factors was tested using a PERMANOVA. Environmental factors, which were selected with the distance-based linear model, were fitted onto an NMDS, which included 160 sites (the number of sites for which all environmental factors were available), using “envfit” in vegan. Pearson correlation between BC dissimilarity and geographic distances (km) were obtained using Mantel tests with the function “mantel” in vegan. Hierarchical agglomerative clustering was based on BC similarities between sites, the Ward’s minimum variance method (Ward D2) and resulting clusters were defined as BCTs (Supplementary Figure 2). Differences of numeric environmental factors among BCTs were determined with ANOVA followed by TukeyHSD *post hoc* test and were based on site-means of environmental factors. Differences of categorical factors, i.e., “biogeographic region” and “land-use type” among clusters were assessed using the Fisher’s exact test and *post hoc* tests were performed with “pairwiseNominalIndependence” with Benjamini-Hochberg corrected *p*-values (rcompanion). Association of OTUs to BCTs and environmental factors were performed with “multipatt” from indicspecies with the point biserial correlation coefficient using the function “r.g” and 10,000 permutations (De Cáceres and Legendre, 2009). Bipartite networks were constructed using the software Cytoscape 3.6.1 (Shannon et al., 2003).

## Results

### Soil Bacterial Community Composition

Sequence analyses of all 1,010 samples yielded 28,474,031 quality filtered bacterial sequences (mean of 28,192 and SD of 10,642 sequences per sample), which formed 48,568 OTUs with a sequence identity threshold of 97% (mean of 3,158 and SD of 1,084 OTUs per sample; Supplementary Table 2). Relative abundances of OTUs ranged from 1.5 × 10^–3^ to 32% per sample. Ten percent of the OTUs (4,933) occurred only at one site and accounted for 0.07% of the sequences. Seven percent of the OTUs (3,323 OTUs) were detected in more than half of the sites and represented 76.5% of the sequences. Only 19 OTUs occurred at all sites and included 4.8% of the sequences. Mean Good’s coverage across all sample was 0.79 (SD 0.06). Ninety-two percent of the OTUs could be taxonomically assigned at the phylum-level and 19.9% at the genus-level. In total 52 phyla were detected, of which 10 included more than 1% of the sequences. The most abundant phylum was Proteobacteria (26.4% of the sequences), followed by Acidobacteria (18.0%), Verrucomicrobia (17.3%), Planctomycetes (10.1%), Chloroflexi (7.8%), Bacteroidetes (5.0%), Actinobacteria (4.2%), Patescibacteria (3.6%), Gemmatimonadetes (1.7%), and Rokubacteria (1.2%).

### Correlation of Soil Bacterial Community Structures With Geographic Distance

Site-specificity of bacterial community structures was assessed based on Bray Curtis (BC) similarities of all samples from each site (distances of 4.6 to 6.6 m) and similarities among different sites based on mean OTU abundances for each site (distances of 7.2 to 345.4 km). Bacterial community structures from each site were more similar, which was revealed by significantly higher mean BC similarities to the respective site-centroids (mean of 0.73, SD 0.07) as compared to those among the centroids from all sites (mean of 0.35, SD 0.15, *t*-test, *p* < 2.2 × 10^–16^). This was further substantiated by a high overall reallocation success of 84.6% (canonical analysis of principal coordinates, CAP) for individual bacterial communities to their site of origin. Mostly four independent soil samples (10 missing samples) were obtained for each site and bacterial community structures, i.e., BC dissimilarities, were determined based on mean OTU abundances for each site. Mean bacterial community structures (based on site) did not correlate with geographic distances (Mantel test, Pearson *r* = 0.08, *p* = 0.0001) revealing a negligible distance-decay relationship of bacterial community structures across Switzerland.

### Soil Bacterial Community Structures in Biogeographic Regions and Land-Use Types

The six distinct Swiss biogeographic regions were represented by different numbers of sites ranging from 8% for the Western Central Alps to 29% for the Northern Alps (Figure 1 and Supplementary Table 1). Furthermore, of the five land-use types, forest accounted for 44% of the sites, alpine grasslands for 18%, arable land for 10%, meadows for 24% and settlements for 4%. The specific environmental conditions of biogeographic regions allow for only certain land-use types, which resulted in different occurrences of land-use types within each biogeographic region (Supplementary Table 3). Significant differences among bacterial community structures were detected for the five different land-use types and for the six biogeographic regions and both factors explained similar amounts of variance if analyzed individually, i.e., 13.6 and 13.0% with 5.3% overlap (overall PERMANOVA, *p* = 0.0001). Pairwise comparisons of the bacterial community structures revealed significant differences among all land-use types and most biogeographic regions except for Eastern and Western Central Alps (pairwise PERMANOVA *p* < 0.05; Supplementary Table 4). These differences were supported by an overall reallocation success of 85.5% and 66.7% (CAP) of bacterial communities to the land-use types and biogeographic regions, respectively (Supplementary Figure 3). Reallocation success to land-use types ranged from 73.3% to 95.6%, except for samples obtained from settlements, which revealed a reallocation success of 33.3%. In addition to the low reallocation success, the nine settlement sites constituted a small and very heterogeneous group of sites with different soil conditions and plant cover. Therefore, these sites were excluded from determining land-use associated OTUs. Reallocation success for samples to the six biogeographic regions ranged from 47.6 to 79.7%.

### Environmental Factor-Based Variance Partitioning of Bacterial Community Structures

**FIGURE 2:**
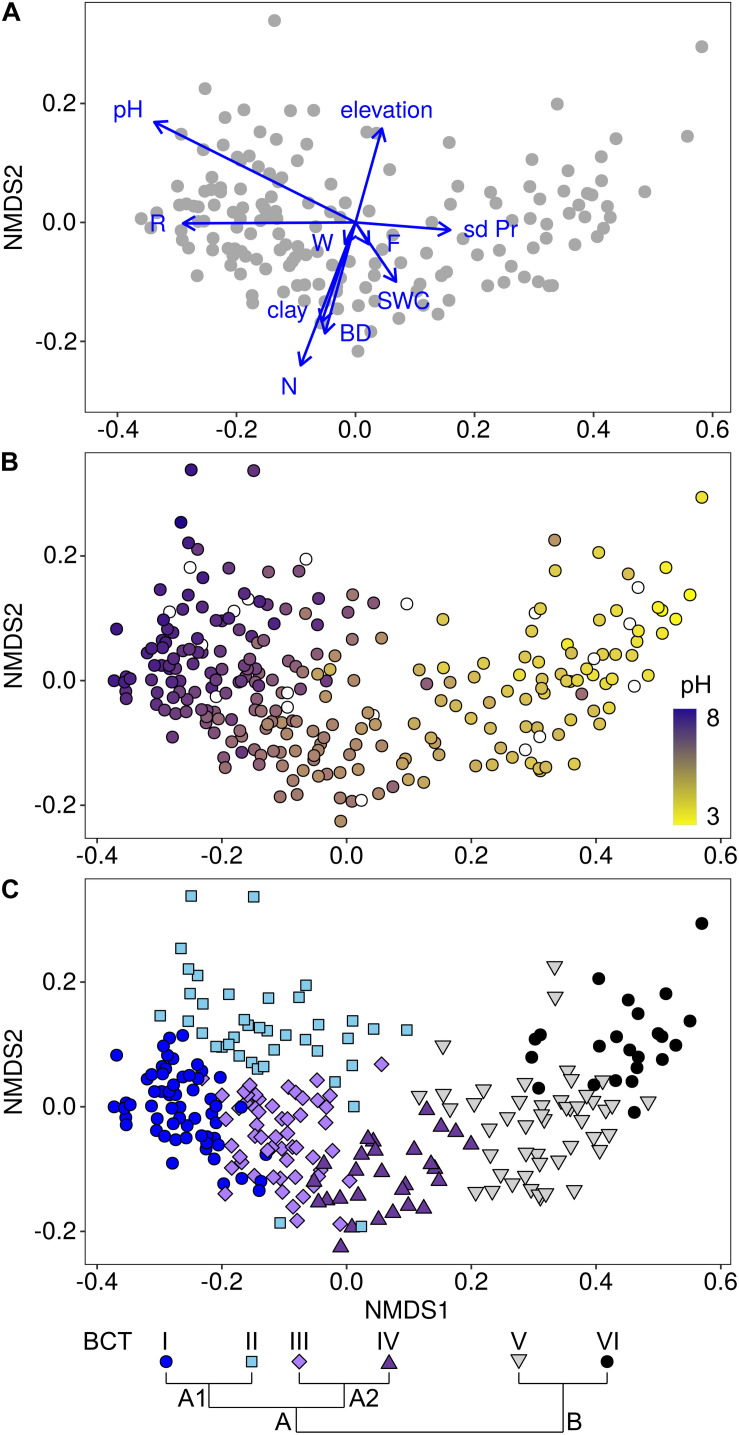
Associations of bacterial communities with environmental factors **(A)**, specifically with soil pH **(B)** and with bacterial community types (BCTs; **C**). The non-metric multidimensional scaling (NMDS) plots were based on Bray-Curtis dissimilarities among 160 sites, for which all environmental factors were available **(A)** and among all 255 sites **(B,C)**. Environmental factors, i.e., pH, elevation, clay content, inter-annual variation in monthly precipitation (sd Pr), soil water content (SWC), soil bulk density (BD) and the plant indicators for soil nutrients (N), soil pH (R), soil moisture (F) and variability of soil moisture (W), which were selected in the linear model (Table 2), were fitted onto the NMDS **(A)**. Length of vectors represent the strength of correlations and they point toward the largest correlation. The color gradient displays the pH gradient **(B)** from high pH in dark purple (maximum pH = 7.8) to low pH in yellow (minimum pH = 2.9). Sites with missing pH data are displayed in white. Associations of BCTs and sites are displayed with colored symbols **(C)**. Pairs of adjacently clustering BCTs included BCT I (dark blue circles; *n* = 60 sites) and BCT II (light blue diamonds; *n* = 40 sites), BCT III (light purple squares; *n* = 56 sites) and BCT IV (dark purple upward pointing triangles; *n* = 28 sites) and BCT V (gray downward pointing triangles; *n* = 48 sites) and BCT VI (black circles; *n* = 23 sites). Clustering of BCTs is shown schematically **(C)** and in detail in Supplementary Figure 2.

Environmental factors that significantly explained variance among bacterial community structures were identified using a distance-based linear model. This allowed to identify ten environmental factors that in total significantly explained 47.9% of the variance in bacterial community structures (*p* < 0.05; Table 2, Figure 2A, and Supplementary Tables 5, 6). Of these factors, pH was most important in structuring bacterial communities by explaining 29.3% of the variance. This importance in shaping the bacterial communities was supported by the gradient representing soil pH in a NMDS plot of mean community structures of each site (Figure 2B). Further, fitting environmental vectors onto the NMDS, which included all 160 sites used for the model selection, revealed that pH and the plant indicator for pH correlated with bacterial community structures in a similar direction along the first, i.e., most important, axis of the NMDS (Figure 2A).

**TABLE 2:** Variance of bacterial community structures explained by environmental factors, land-use type, and biogeographic region.

Environmental factor^a^	Pseudo-F	Explained variance [%]	Cumulative explained variance [%]
Soil pH	65.6	29.3	29.3
Plant ind.^b^ for nutrients	14.5	6.0	35.3
Elevation	7.3	2.9	38.2
Clay content [%]	5.5	2.1	40.3
Bulk density [g dried fine^c^ soil/cm^3^]	4.5	1.7	42.0
Soil water content [%]	4.5	1.6	43.6
Plant ind.^b^ for pH	3.8	1.4	45.0
Plant ind.^b^ for soil moisture	3.1	1.1	46.1
Plant ind.^b^ for variability of soil moisture	2.6	0.9	47.0
SD^d^ of inter-annual precipitation [mm]	2.5	0.9	47.9
Land-use type^e^	2.4	3.3	51.2
Biogeographic region^e^	1.7	2.8	50.7

*^*a*^Factors were ranked in a step-wise forward selection using the Akaike information criterion with a *p*-value larger than 0.05 as exclusion criterion for the linear model, which was based on the complete dataset available for 160 sites (Supplementary Table 6).*

*^*b*^Plant indicator.*

*^*c*^Sieved to 2 mm.*

*^*d*^Mean standard deviation of monthly precipitation sums from between 1981 and 2010.*

*^*e*^Land-use type and biogeographic region were added individually to the model after taking into account environmental factors.*

The second most explanatory environmental factor in the model was the plant indicator for nutrients with an *R*^2^ value of 6.0%, which mainly reflects nitrogen and phosphorus availability in soils. The third factor was elevation (*R*^2^ = 2.9%; Table 2), with a strong negative correlation with temperature (*r* = −0.99). Elevation was followed by clay content (*R*^2^ = 2.1%) and bulk density (*R*^2^ = 1.7%). All four factors, the plant indicator for nutrients, elevation, clay content and bulk density were represented by vectors pointing in the direction of the second axis of the NMDS (Figure 2A). The subsequent factors in the model were soil water content (*R*^2^ = 1.6%), the plant indicator for pH (*R*^2^ = 1.4%), the plant indicator for soil moisture (*R*^2^ = 1.1%) and the plant indicator for variability of soil moisture (*R*^2^ = 0.9%), as well as the variability of monthly precipitation (*R*^2^ = 0.9%). Furthermore, “land-use type” and “biogeographic region” still explained small but significant (*p* < 0.05) amounts of variance, i.e., 3.3% and 2.8%, when the other environmental factors were first taken into account (Table 2).

### Bacterial Community Types in Swiss Soils

**FIGURE 3:**
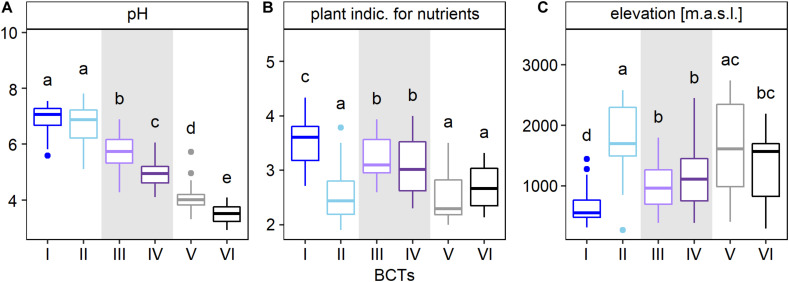
Characterization of bacterial community types (BCTs) based on the environmental factors pH **(A)**, plant indicator for nutrients **(B)**, and elevation **(C)**. The boxplots show median (middle horizontal bar), 75th and 25th percentile (upper and lower limit of the box), values up to 1.5 times the interquartile range above and below the box (whiskers) and values larger than 1.5 times the interquartile range (dots). Number of sites that were available for each factor were 236 for pH and 255 for the plant indicator for nutrients and elevation. BCT I to VI were defined using hierarchical clustering based on Bray-Curtis dissimilarities of mean OTU abundances for each site and the Ward’s minimum variance method (Ward D2). Pairs of adjacently clustering BCTs, i.e., BCT I (dark blue) and II (light blue), BCT III (light purple) and IV (dark purple), and BCT V (gray) and VI (black) were highlighted with a white or gray background. Factors were ordered according decreasing effect size (F-statistic) and the three factors with the largest differences among BCTs, omitting the plant indicator for pH because of its similarity with soil pH, are shown (ANOVA, Supplementary Table 7). Lower case letters represent significant differences (Tukey test, *p*-value < 0.05). Plots for the remaining 20 factors are shown in Supplementary Figure 4.

Cluster analysis was used to assess the main structure types of soil bacterial communities at the 255 sites across Switzerland. This analysis divided bacterial community structures into two highest order clusters A and B (Figure 2C and Supplementary Figure 2). Cluster A formed four sub-clusters at the third clustering level, while cluster B formed two sub-clusters. Each of these six sub-clusters was defined as a bacterial community type (BCT) that also represented distinct groups in the NMDS ordination (Figure 2C). Furthermore, environmental factors differed among sites, at which different BCTs were detected, supporting the clustering of the BCTs (Figure 3, Supplementary Figure 4, and Supplementary Table 7). Soil pH was the factor, which differed most among the six BCTs (overall ANOVA, *F* = 241.5, *p* = 6.03E-88). Pairwise comparisons revealed that soils harboring BCT I or II did not differ in pH (with a mean pH 6.8 and 6.7), but the mean pH differed significantly across soils, which harbored the other BCTs, i.e., BCT III (mean of 5.8), BCT IV (mean of 4.9), BCT V (mean of 4.0), and BCT VI (mean of 3.6). Soils that included BCTs I or II had a pH close to neutral, however, they differed in twelve other environmental factors (Figure 3 and Supplementary Figure 4). Interestingly, BCT II occurred at sites with bacterial communities better adapted to lower soil nutrient values as revealed by the plant indicator N (mean of 2.5), higher elevation (mean 1,823.6 m.a.s.l.), higher carbon to nitrogen ratio (mean 18) and a lower clay but higher sand content as compared to sites harboring BCT I. Also, the factors differing significantly and most strongly among sites at which the low pH BCT V or VI occurred were organic carbon, total nitrogen and the carbon to nitrogen ratio (Supplementary Figure 4). BCT III and IV differed in their preference for soil pH but for none of the other environmental factors.

### Association of Soil Bacterial Community Types With Biogeographic Regions and Land-Use Types

**FIGURE 4:**
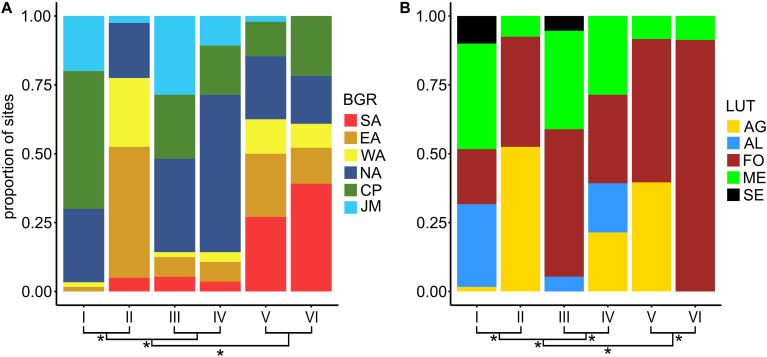
Proportion of sites from the six biogeographic regions (BGR; **A**) and the five land-use types (LUT; **B**) among the six bacterial community types (BCTs). BCT I to VI correspond to clusters based on Bray Curtis dissimilarities based on mean OTU abundances for each site and the Ward’s minimum variance method (Ward D2) mean OTU abundances per site. Clustering of BCT I to VI is shown at the bottom of the barplots. Asteriks at branchings denote significantly (Fisher exact test, adjusted *p*-value < 0.05) different composition of the BGRs, i.e., Jura Mountains (JM), Central Plateau (CP), Northern Alps (NA), Eastern Central Alps (EA), Western Central Alps (WA), and Southern Alps (SA), or of the LUTs, i.e., alpine grassland (AG), arable land (AL), forest (FO), meadow (ME), and settlement (SE).

Bacterial community types were significantly associated to biogeographic regions as well as land-use types (overall Fisher exact test, *p* < 0.001) indicating that BCTs occurred in different land-use types and different biogeographic regions (Figure 4). However, none of the six BCTs was found at only one land-use type or biogeographic region. Various geographic patterns were detected for BCT I to IV (Supplementary Figure 5). BCT I and II occurred at sites with neutral soil pH, however BCT I was detected almost exclusively (96.7%) north of the main Alpine ridge (including Jura, Central plateau and Northern Alps) and contained all land-use types. In contrast, BCT II occurred at soils with neutral soil pH from the Central Alps with some sites (25.0%) from the Northern and Southern Alps but none from the Central Plateau and as a consequence contained many alpine grasslands in addition to forest and a few meadows but no arable land. Similar to BCT I, but less consistent, BCT III and IV were mostly detected north of the Alpine ridge (for both 85.7% of the respective sites). Most of the sites from the Southern Alps (79%) harbored BCT V and VI. BCT VI occurred mainly at forest sites (91.3% of the sites) and few meadows (8.7%), whereas BCT V occurred at sites from forest (52.1%), alpine grassland (39.6%), and meadows (8.3%).

### Environmental Factor and BCT-Based Partitioning of Indicator OTUs

**FIGURE 5:**
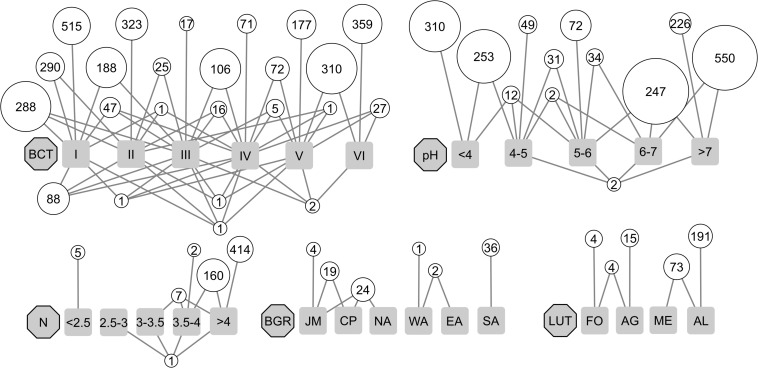
Associations of OTUs to bacterial community types (BCT I to VI; in total 2,931 OTUs) and different environmental factors such as soil pH (pH; in total 1,788 OTUs) and the plant indicator value for soil nutrients (N; in total 589 OTUs) as well as biogeographic region (BGR; in total 86 OTUs), including Jura Mountains (JM), Central Plateau (CP), Northern Alps (NA), Eastern Central Alps (EA), Western Central Alps (WA), and Southern Alps (SA) and land-use type (LUT; in total 287 OTUs), including forest (FO), alpine grassland (AG), meadow (ME), and arable land (AR). Factors are represented by gray octagons and their different levels by gray squares. Levels of pH and N include open intervals at the left end and closed intervals at the right end. OTUs associated to the same level or levels are represented by white circles. Numbers in the circles represent the number of included OTUs while their diameters represent the total relative abundance of associated OTUs. The white circles of each factor are arranged in “rows” with the upper most “row” including OTUs associated to a single level. The lower “rows” included OTUs associated to an increasing number of factor levels. Additional factors that revealed OTU-associations are shown in Supplementary Figure 6.

Indicator species analyses were performed in order to identify OTUs that significantly associated to environmental factors included in the linear model, biogeographic regions, land-use types, or BCTs (point biserial correlation coefficient rPB > 0.5 and BH adjusted *p*-value < 0.05). In total, 7,385 associated OTUs were identified (Table 3). Of these, 4,081 were significantly associated to environmental factors, 287 to land-use types and 86 to biogeographic regions. Of the 10 environmental factors considered for associations clay content and the plant indicators for moisture and variability of moisture yielded no significantly associated OTUs (Table 3). With 1,788 and 1,406 associated OTUs, the highest numbers derived from the factors pH and plant indicator for pH and 871 were associated to both (Table 3). BCTs yielded a total of 2,931 OTUs (6.0% of all) that were significantly associated to one or more BCTs and together they accounted for 58.7% of the total sequence abundance (Figure 5 and Table 3). If all redundant associated OTUs were removed, a total of 3,998 remained, revealing 3,387 OTUs, which were associated to more than one factor. The seven environmental factors that yielded associated OTUs jointly contributed 2,867 non-redundant OTUs, while biogeographic region yielded 16 and land-use type 66. For BCTs, 1,049 associated OTUs remained that accounted for 11.8% of the total sequence abundance (Table 3). This indicated that 73.8% of the BCT-associated OTUs were also associated to one or more environmental factor, land-use type, or biogeographic region, while 26.2% of them were only associated to BCTs. Bipartite network analyses of the environmental factors revealed that most of the 1,788 OTUs that were associated to pH were either associated to acidic soil conditions (pH ≤ 5, 34.2%) or to weakly acidic to weakly alkaline soil conditions (pH > 5, 63.1%; Figure 5). Of the 586 OTUs associated to the plant indicator for nutrients 98% correlated with nutrient rich soils (*N* > 3.5 on a scale from 0 to 5). Of the 287 OTUs associated to land-use types, 92.0% were associated to arable land or both arable land and meadows. Most of the 86 OTUs associated to biogeographic regions were either associated to the Southern Alps (30.2%) or to regions north of the alpine ridge, i.e., Jura, Central Plateau and Northern Alps (51.2%; Figure 5). Most of the OTUs associated to variability of precipitation (i.e., 99.3%), elevation (100%), bulk density (94.3%), and soil water content (100%) were associated to the lowest or the highest levels, however, these associations were less pronounced, i.e., with a lower maximal correlation coefficient, than the ones for the above-mentioned factors (Supplementary Figure 6 and Table 3). Of the 2,931 OTUs associated to BCTs 1,462 were associated to single BCTs and this number decreased if two (991 OTUs), three (385 OTUs), four (92 OTUs), or five (1 OTU) BCTs were considered (Figure 5). The highest numbers of OTUs associated to single BCTs were 515, 323, and 359 OTUs to BCT I, II, and VI, while BCT III, IV, and V included only 17, 71, and 177 associated OTUs.

**TABLE 3:** Associations of OTUs to environmental factors, biogeographic regions, land-use types and bacterial community types (BCTs).

Environmental factors^a^	Total^b^		Non-redundant^c^				Maximum of r_*PB*_^g^
		
	Associated OTUs [N]^d^	Rel. abund. [%]^e^	Associated OTUs [N]^d^	Rel. abund. [%]^e^	Cumul.^f^ [N]	Cumul.^f^ [%]	
pH	1,788	45.6	1,788	45.6	1,788	45.6	0.78
Plant ind.^h^ for pH	1,406	30.9	535	3.4	2,323	49.0	0.79
Plant ind.^h^ for nutrients	589	7.0	454	1.5	2,777	50.5	0.78
SD of monthly precipitation^i^	148	3.8	39	0.1	2,816	50.7	0.68
Bulk density	70	1.3	31	0.5	2,847	51.1	0.62
Elevation	47	3.1	10	0.2	2,857	51.3	0.60
Soil water content	33	0.7	10	0.1	2,867	51.4	0.57
Biogeographic region	86	4.4	16	0.3	2,883	51.7	0.63
Land-use type	287	6.4	66	0.9	2,933	52.6	0.74
Bacterial community types	2,931	58.7	1,049	11.8	3,998	64.4	0.81

*^*a*^Environmental factors were ordered according to the number of associated OTUs, followed by biogeographic region, land-use type and bacterial community types.*

*^*b*^Associated OTUs to each factor individually and their relative abundance.*

*^*c*^Associated OTUs to pH and number of associated OTUs to each factor in addition along the column in the table, thereby excluding redundant OTUs.*

*^*d*^Selection of OTUs with a Benjamini and Hochberg adjusted *p*-value below 0.05 and r_*PB*_ larger than 0.5 based on all sites which were available for each factor.*

*^*e*^Relative sequence abundance.*

*^*f*^Cumulative.*

*^*g*^r_*PB*_, point biserial correlation coefficient, which is used in case one of the variables is dichotomous.*

*^*h*^Plant indicator.*

*^*i*^Inter-annual standard deviation of monthly precipitation sums between 1981 and 2010.*

### Taxonomic Classification of OTUs Associated to Strongest Drivers

Taxonomic classification to the genus level was performed for OTUs associated to strong environmental drivers of particular interest, i.e., pH, the plant indicator for nutrients, land-use types and biogeographic regions (Supplementary Table 8). Taxonomic classification yielded 169 genera and 215 higher-level taxa. These taxa were assigned to 30 different phyla and 23 of them included taxa associated to different levels of pH, the plant indicator for nutrients and land-use type while the remaining seven phyla included only one or two taxa. For example, the phylum of Acidobacteria contained twelve taxa that were associated to either low or high pH. OTUs of the genera *Acidipila, Granulicella, Occallatibacter*, and Candidatus *Koribacter* as well as the acidobacterial orders Subgroup 2 and 13 were associated to acidic conditions (pH ≤ 5). OTUs of the acidobacterial genera *Paludibaculum* and *Stenotrophobacter* and Subgroup 10 as well as the acidobacterial classes Subgroup 9, 18, and 25 were associated to pH above 6. The most frequent taxon of the associated OTUs was the acidobacterial class Subgroup 6, of which most members were associated to slightly acidic to slightly alkaline conditions (7.8 ≤ pH > 6). Most of the elven associated OTUs of the phylum Firmicutes, which included the eight genera *Bacillus, Caldicoprobacter, Tepidimicrobium, Hydrogenispora, Paeniclostridium, Romboutsia, Terrisporobacter*, and *Turicibacter*, revealed a similar distinct ecological association (Supplementary Table 8). All of them were associated to high nutrient content and/or to managed sites and none of them to pH. Furthermore, taxa including OTUs associated to high nitrogen availability were from the genera *Nitrospira* (Nitrospirae), *Nitrosospira* (Proteobacteria) and *Luteolibacter* (Verrucomicrobia). Nine genera, which were associated to land-use types or biogeographic regions but not to pH or the plant indicator for nutrients were found. Of these, six included OTUs which were associated to arable land, i.e., *Dechlorosoma* (Gammaproteobacteria), *Massilia* (Gammaproteobacteria), *Marmoricola* (Actinobacteria), *Oryzihumus* (Actinobacteria), *Proteiniborus* (Firmicutes), and *Tepidisphaera* (Planctomycetes), while the genus *Diplosphaera* (Verrucomicrobia) was associated to arable land and grassland. Two genera were associated to biogeographic regions, i.e. *Hyphomicrobium* (Alphaproteobacteria) to the Eastern and Western Central Alps and Candidatus *Jidaibacter* (Alphaproteobacteria) to the Southern Alps. OTUs, which were exclusively associated to BCTs and not to any other of the environmental factors considered, i.e., an additional of 1,049 OTUs representing 35.8% of the associated OTUs, originated from 24 phyla, 99 families and 96 genera, and revealing a high taxonomic diversity that reacted to this far unassessed environmental factors that shape soil bacterial community structures.

## Discussion

The goal of this study was to provide a detailed analysis of soil bacterial communities across the complex landscape of Switzerland and an assessment of factors explaining their structures and compositions. To achieve this goal, 255 locations situated on a regular grid across the entire country were sampled (Figure 1). A total of 1,010 soil samples were analyzed and bacterial communities were determined using metabarcoding based on PCR amplification of a fragment of the 16S rRNA gene.

### Geographic Distribution of Bacterial Community Structures

Switzerland encompasses a high complexity of distinct biogeographic regions with the Jura Mountains, the Central Plateau, the Northern Alps, the Eastern and Western Central Alps as well as the Southern Alps. If considered separately, a significant proportion of the variance of bacterial community structures was explained by the factor “biogeographic region” (13.0%). Although bacterial community structures revealed geographic distribution patterns across Switzerland, a distance-decay pattern was not found (*r* = 0.08). Environmental factors, particularly pH, influenced bacterial community structures to a much greater extent and all environmental factors together explained 47.9% of their variance. Therefore, selection by environmental factors appeared to play a major role in the biogeographic distribution of bacterial communities across Switzerland. A distance-decay relationship for bacterial community structures has been reported at the regional scale, spanning distances of several 100s of km (Ranjard et al., 2013; Barnett et al., 2019; Feng et al., 2019). However, these studies also found that the distance-decay relationship was largely driven by environmental selection, supporting the importance of environmental selection. A further reason why we did not detect a distance-decay relation might be that in a mountainous area such as Switzerland, geographic distance is less important than fragmentation of the area where, for example, elevational and geological differences among sites become more important. However, our finding of no distance-decay relationship, does not exclude the possibility that some bacterial taxa followed a distance-decay relationship and others were driven by selection factors. An insightful way to study these processes would be to assess the distribution of single bacterial taxa or genotypes across regions and time.

### Bacterial Communities in Different Land-Use Types

If considered separately, the factor “land-use type,” including forest, alpine grassland, meadow, arable land and settlement, explained 13.6% of the variance of bacterial community structures. This is in accordance with a study that has assessed the effect of “land-use types” (forest, grassland and arable land) on bacterial community structures across a European transect and has reported that “land-use” explained 15% of the variance (Szoboszlay et al., 2017). In the present study, land-use types were also linked to specific geographic conditions, since they depend on specific soil properties as well as climatic factors (Jaffuel et al., 2018). Therefore, specific land-use types predominantly occur in certain biogeographic regions (Supplementary Table 3). It is difficult to disentangle and rank the specific impact of these two factors on soil bacterial communities since they explained similar sized proportion of the variance (13.6 and 13.0%, respectively) with an overlap of 5.3%. Furthermore, land-use types differed in their plant cover, e.g., grassland, forest, and arable land, disturbance frequency, e.g., managed arable land compared to less managed alpine grassland and forest, as well as management intensity, e.g., quantity and type of nutrient input, which all have been shown to affect bacterial communities (Hartmann et al., 2015; Prober et al., 2015). Overall, of the 48,568 OTUs detected in this study 287 OTUs (0.6%) were associated to land-use and of those, 264 were associated to the managed land-use types arable land and meadow, while only 23 were associated to the less managed types forest and alpine grassland. This suggested that certain bacterial taxa are introduced or favored by management, despite the considerable variability of environmental conditions within the land-use types. In a study comparing the impact of different management regimes on bacterial communities, the authors have reported that the impact of farm yard manure was very strong and resulted in the presence of specific bacterial OTUs (Hartmann et al., 2015). Overall, the effect of land-use on bacterial communities in Swiss soils provided support for the sensitivity of soil bacteria to anthropogenic impact, regardless of the interaction with environmental factors.

### Environmental Factors Shaping Bacterial Communities

As reported in numerous studies ranging from experimental fields to the global scale, soil pH was the dominant factor for explaining variance of bacterial community structures (e.g., Fierer and Jackson, 2006; Lauber et al., 2009; Bahram et al., 2018). In the present study, soils covered a pH range from 2.9 to 7.8 and the importance of pH became also evident by contributing most to explaining the variance of bacterial communities (Table 2) as well as by the large number (1,788 OTUs) of bacterial taxa, which were specifically associated to different levels and ranges of pH (Figure 5). The bipartite network analysis revealed that most OTUs that were indicative for pH, were associated to soils with a pH either below 5 (three clusters with 612 OTUs) or above 5 (five clusters with 1,129 OTUs). The separation at a pH of about 5 was only bridged by four clusters containing 47 OTUs. This suggested that bacterial taxa were mainly adapted to pH conditions either below pH 5 or above pH 5. Similarly, pH 5 was identified as one of the pH related splits separating 16 microbial habitats across France (Karimi et al., 2020). Comparison of the taxonomy of the pH-associated OTUs revealed that they were not conserved at the phylum level, i.e., taxa of one phylum could be associated to different pH levels or not be associated at all, which precluded drawing conclusions on pH-associations at the phylum level (Naether et al., 2012). The pH preference of taxa from the phylum *Acidobacteria* corresponded largely to that reported in a comprehensive review (Kielak et al., 2016). For instance, taxa of the acidobacterial class *Acidobacteriia*, including subgroups 2 and 13 as well as the genera *Acidipila, Granulicella* and *Occallatibacter*, were associated to acidic soil conditions (Supplementary Table 8). In the present study, the most frequent of all pH-associated taxa was the acidobacterial subgroup 6, which occurred almost exclusively in soil conditions close to neutral pH. In a previous study, the acidobacterial subgroup 6 has been reported to show higher abundances in neutral or alkaline soils as compared to acidic soils and it was among the most abundant taxa of the phylum *Acidobacteria* in grasslands of the Western Swiss Alps (Yashiro et al., 2016) as well as in soils across an elevation gradient in the Shennongjia mountains in China (Zhang et al., 2014).

Soil nutrient content was either directly assessed by determining total carbon, organic carbon, and total nitrogen contents or indirectly by using the plant indicator value for nutrients (Landolt, 2010). The plant indicator value for nutrients was the second most important factor by explaining 6% of the variance of bacterial community structures, while the direct measures were excluded due to lack of significance. To our knowledge, the plant indicator for nutrients has not yet been applied to assess effects of nutrient availability on soil bacterial community structures. As it is based on the plant species growing at the site, it may characterize the bioavailable part of soil nutrients (Ewald et al., 2013) not only for plants but also for bacteria. A high number of associated OTUs (574) that correlated with high nutrient availability derived from this plant indicator (*N* > 3.5), while only few were associated to a low nutrient availability. All 11 associated taxa of the phylum *Firmicutes* were associated to high nutrient availability and some of them were associated to arable land or meadows. This suggested that taxa of the *Firmicutes* respond to high soil nutrient content. This is in accordance with two studies in which the increase in nutrients lead to an increase of the phylum *Firmicutes*. A 38% increase in abundance of *Firmicutes* has been found after the addition of bovine urine to soil (O’Callaghan et al., 2010). Furthermore, an increase from 5 to 40% in relative abundance of *Firmicutes* has been observed in soils, which had experienced a 240 fold increase in dissolved organic matter after a rapid artificial increase of pH from 4.7 to 8.3 (Anderson et al., 2018). Results from the present study suggested that Firmicutes react rather to nutrients than to pH. The genera *Nitrospira* (phylum *Nitrospirae*) as well as *Nitrosospira* (phylum *Gemmaproteobacteria*) were indicative for high nutrient availability in the present study. These associations may be explained by their ecological role, as both genera are involved in nitrification (Prosser et al., 2014; Daims et al., 2015). Association of the genus *Luteolibacter* (phylum *Verrucomicrobia*) to high nutrient availability is less well described, as the genus includes chemo-organoheterotrophic taxa, which were isolated from a broad range of habitats such as soils, the deep sea as well as plants and are able to degrade simple sugar and complex protein substrates (Pascual et al., 2017). The taxonomic identification of associated OTUs allowed to demonstrate and in some cases confirm the preference for high nutrient availability of certain taxa and allowed to assess their potential ecological roles or preferences. However, this analysis is still limited, because 56.0% of the taxa (Supplementary Table 8) remained without a classification at the genus-level and due to the lack of knowledge on the ecological preferences of many taxa.

Elevation correlated strongly and negatively with mean annual temperature and the plant indicator for nutrients (Supplementary Table 9), suggesting that elevation-related, nutritional and climatic factors similarly affect bacterial communities. Differences of bacterial communities across elevational gradients has previously been reported and similar to the present study, elevation correlated with other environmental factors such as temperature, precipitation and/or plant cover (Singh et al., 2014; Peay et al., 2017). In addition, no elevation-effect on bacterial communities has been found in a study with little differences in pH and moisture along the elevational gradient from 200 to 3,450 m (Fierer et al., 2011). This makes it difficult to generalize effects of elevation on bacterial communities and suggests that effects of elevation rather represent a combination of effects related to other environmental factors.

### Bacterial Community Types

In order to better understand the distribution, diversity, and ecology of bacterial community structures, the 255 soil bacterial communities were divided into six clusters based on similarities of their structures and were designated as bacterial community types (BCT I to VI). BCTs have most likely developed over some time and therefore included bacterial taxa adapted to certain environments. Similarly, the framework of defining plant community types has been used as the basis of phytosociological approaches and the formation of plant based habitat types. Well-described examples are the system of plant based habitat types across Switzerland (Delarze et al., 2015) or forest habitat types in different states of the U.S., for instance in Montana (Pfister and Arno, 1980). Recently, 16 microbial habitats of bacterial communities have been defined across France (Karimi et al., 2020). These microbial habitats were based on environmental factors, which best discriminate similar bacterial communities, i.e., pH, land-use, carbon to nitrogen ratio, organic carbon, and mean annual temperature. In contrast, the here presented approach defines BCTs entirely based on similarities of bacterial community structures and subsequently their habitat preferences were determined. BCT I and BCT II were both associated to soils with a neutral pH (mean pH of 6.8 and 6.7, respectively), but differed in their geographic distribution. This indicated that different soil bacterial communities occur in different geographic patterns despite a similar pH preference. BCT III and IV also occurred predominantly north of the alpine ridge, while BCT V and VI occurred in acidic soils and were not detected in the Jura. Distribution patterns of BCTs may become more evident if larger sampling areas will be considered. For instance, BCT VI occurred almost exclusively in strongly acidic forest soils (Figure 4) and therefore its distribution may correspond to the distribution of acidic forest soils, which also show structured patterns at the global scale (Brunner and Sperisen, 2013). Furthermore, BCT V and VI, which were detected in acidic soils (mean pH of 4.0 and 3.6), included large ranges of elevations but mainly differed in organic carbon and total nitrogen contents as well as their ratio, which only became evident when considering BCTs. When soil preferences of certain BCTs were compared to the 16 microbial habitat types described across France (Karimi et al., 2020), striking similarities were observed. Most obvious, pH was an important factor that caused four separations, including the highest order separation of the microbial habitat types in France. Of the extremely acidic microbial habitats (pH < 5) three were associated to forests and one to grasslands and all were differentiated by medium to high carbon to nitrogen ratio, which resembled the preferred environmental conditions of BCT V and VI. The acidic and slightly acidic microbial habitats were differentiated by land-use and soil organic carbon. In contrast, the BCTs with corresponding pH range (III and IV) ncluded several different land-use types. Furthermore, no counterpart of BCT I and II, which were differentiated by sites occurring in different biogeographic regions with different elevation and nutrient content, were observed in the French system. Also, the alkaline complex of French microhabitats was not detected in the present study. In summary, BCTs showed similarities to the French microbial habitats especially at extremely low pH. Overall, the analyses of BCTs revealed distribution patterns and additional factors, such as nutrient availability, that differentially shaped soil bacterial communities among sites. The analysis of soils from additional sites, characterizing their bacterial communities and comparing them to the BCTs will be important to validate the six BCTs described here for different Swiss soils. This may also be helpful to confirm their association and relation to environmental factors or possibly allow defining new or more refined BCTs. It would then be interesting to map distributions of BCTs at the global scale. BCTs could be associated to soil functions, possibly indicating soil quality or fertility. Therefore, BCTs may represent units that could be included in soil monitoring programs and be traced over time. For instance, the conversion of the bacterial community at a site from one BCT into another could be assessed and possibly linked to a change in environmental conditions. Thus, BCTs may provide a new, empirically defined framework for soil bacterial communities, which may facilitate our understanding of the complex distribution and habitat preferences of soil microorganisms, populations, or communities.

### Associations of OTUs to Bacterial Community Types and Environmental Factors

In total, 2,931 OTUs, i.e. 6% of all, were associated to one or more BCTs. These OTUs formed 24 groups, and included 58.7% of the total relative abundance, revealing that a large part of the sequences was associated to and therefore contributed to structuring the BCTs. Of the BCT-associated OTUs, 26.2% were exclusively associated to BCTs and their distributions may result from their preferences for unassessed environmental factors. Therefore, taxonomic analysis of these OTUs paired with knowledge of their ecology may provide helpful insights into hitherto unaccounted environmental factors. Furthermore, the bipartite network of the BCT-associated OTUs revealed relations among BCTs. Almost half of the BCT-associated OTUs (1,462) were indicative for single BCTs (Figure 5). The remaining OTUs were associated to multiple mostly consecutive BCTs revealing the relation among them as shown by the cluster analysis (Supplementary Figure 2). Only three groups with 188, 47, and 1 OTUs revealed a non-consecutive behavior and all of them bridged from BCT I to BCT III skipping BCT II. This indicated a specific nature of BCT II, which was characterized by occurrence in neutral soils with low nutrient availability at high elevations and may explain the separation of neutral soil microbial communities into two distinct BCTs (Figure 2). On the other hand, there were two consecutive groups of OTUs that included BCT-II and bridged BCTs I-III (288 OTUs) or BCTs I-IV (88 OTUs). This provided support for the similarities of these BCTs and their closer clustering in branch A (Figure 2). This could be explained by their preference for pH above 5 (Figure 3), which matches the finding of pH associated OTUs, where pH 5 was identified as division point of associated OTUs. Taken together, the bipartite network of the BCT-associated OTUs displayed relations among BCTs, identified BCT-characteristic OTUs and revealed 1,049 OTUs with potential information to derive environmental factors that have not been assessed in this study but may represent important for bacterial communities. In addition, the substantial proportion of unexplained variance in bacterial community structures suggested missing explanatory factors. Unaccounted abiotic and biotic factors include the presence of specific micronutrients (Navarrete et al., 2013), soil organic carbon quality (Yashiro et al., 2016; Szoboszlay et al., 2017), redox potential (Lipson et al., 2015), interactions with other soil organisms (Wardle, 2006), or specific soil management differences (Hartmann et al., 2015). More detailed consideration of such factors will help to gain a more profound understanding of their interactions with soil bacterial community structures.

## Conclusion

Our analyses of bacterial communities across Switzerland, with its complexly structured mountainous landscape that is confined to a relatively small area, revealed distinct geographic distribution patterns that differed from a distance-decay relation. Environmental selection was identified as the main driver of bacterial community structures, which was substantiated by the finding that land-use types as well as biogeographic regions explained only little variance of bacterial community structures. We also found that the plant indictor for nutrients was, after soil pH, the second best predictor for bacterial community structures and a better predictor than chemically determined soil nutrients. This suggested that biological proxies for soil nutrient availability may provide an accurate assessment of nutrient availability for soil bacteria. The number of OTUs associated to different environmental factors and their levels, i.e., levels of pH, revealed details on how these may affect bacterial community structures. For instance, pH-associated OTUs largely divided into clusters that associated to soils with a pH either below or above five. In addition, more OTUs were associated to soils with higher nutrient availability. Finally, we proposed BCTs as a complementary framework for studying soil bacterial communities. This allowed the association of BCTs to their habitat preferences regarding environmental factors, biogeographic regions, and land-use types. These revealed further associations and distinctions otherwise undetected. For instance, certain BCTs were absent from certain biogeographic regions revealing distinct geographic distribution patterns. The bipartite networks revealed OTUs, which were exclusively associated to BCTs and which hold potential to identify hitherto unassessed environmental factors that are important for shaping soil bacterial communities.

## Data Availability Statement

The datasets presented in this study can be found in online repositories. The names of the repository/repositories and accession number(s) can be found at: https://www.ncbi.nlm.nih.gov/genbank/ with the project number PRJNA633766.

## Author Contributions

JM analyzed, computed, and interpreted the data, designed the figures, and wrote the manuscript. FW made substantial contributions to the interpretation of the data and the writing process, provided resources in the molecular laboratory, and was responsible for acquisition of funding. RM contributed to the development of the manuscript, was responsible for acquisition of funding, and provided resources in the soil laboratory. TR and LK were responsible for sampling design and organization of sampling, provided plant data and site-specific data, and were involved in the interpretation of the data. DW provided data on soil factors and was responsible for the soil database. PC provided and processed data on climate. All authors read and approved the final manuscript.

## Conflict of Interest

LK and TR were employed by the company Hintermann & Weber AG. The remaining authors declare that the research was conducted in the absence of any commercial or financial relationships that could be construed as a potential conflict of interest.

## References

[B1] Anderson C. R. Peterson M. E. Frampton R. A. Bulman S. R. Keenan S. Curtin D. (2018). Rapid increases in soil pH solubilise organic matter, dramatically increase denitrification potential and strongly stimulate microorganisms from the Firmicutes phylum. *PeerJ* 6:e6090. 10.7717/peerj.6090 30581677 PMC6295159

[B2] Bahram M. Hildebrand F. Forslund S. K. Anderson J. L. Soudzilovskaia N. A. Bodegom P. M. (2018). Structure and function of the global topsoil microbiome. *Nature* 560 233–237. 10.1038/s41586-018-0386-6 30069051

[B3] Barnett S. E. Youngblut N. D. Buckley D. H. (2019). Soil characteristics and land-use drive bacterial community assembly patterns. *FEMS Microbiol. Ecol.* 96:fiz194. 10.1093/femsec/fiz194 31834372

[B4] Brunner I. Sperisen C. (2013). Aluminum exclusion and aluminum tolerance in woody plants. *Front. Plant Sci.* 4:172. 10.3389/fpls.2013.00172 23781222 PMC3679494

[B5] Bühler C. Roth T. (2011). Spread of common species results in local-scale floristic homogenization in grassland of Switzerland. *Divers. Distrib.* 17 1089–1098.

[B6] Bürgmann H. Pesaro M. Widmer F. Zeyer J. (2001). A strategy for optimizing quality and quantity of DNA extracted from soil. *J. Microbiol. Methods* 45 7–20. 11295193 10.1016/s0167-7012(01)00213-5

[B7] Chemidlin Prévost-Bouré N. Dequiedt S. Thioulouse J. Lelièvre M. Saby N. P. A. Jolivet C. (2014). Similar processes but different environmental filters for soil bacterial and fungal community composition turnover on a broad spatial scale. *PLoS One* 9:e111667. 10.1371/journal.pone.0111667 25365044 PMC4218796

[B8] Clarke K. R. Gorley R. N. (2015). *PRIMER v7: User Manual/Tutorial.* Plymouth: PRIMER-E.

[B9] Daims H. Lebedeva E. V. Pjevac P. Han P. Herbold C. Albertsen M. (2015). Complete nitrification by *Nitrospira* bacteria. *Nature* 528 504–509. 10.1038/nature16461 26610024 PMC5152751

[B10] De Cáceres M. Legendre P. (2009). Associations between species and groups of sites: indices and statistical inference. *Ecology* 90 3566–3574. 10.1890/08-1823.1 20120823

[B11] Delarze R. Gonseth Y. Eggenberg S. Vust M. (2015). *Lebensräume der Schweiz: Ökologie - Gefährdung - Kennarten.* Bern: Ott Verlag.

[B12] Dürr B. Zelenka A. (2009). Deriving surface global irradiance over the Alpine region from METEOSAT second generation data by supplementing the HELIOSAT method. *Int. J. Remote Sens.* 30 5821–5841.

[B13] Edgar R. C. (2013). UPARSE: highly accurate OTU sequences from microbial amplicon reads. *Nat. Methods* 10 996–998. 10.1038/nmeth.2604 23955772

[B14] Ellenberg H. Weber H. E. Düll R. Wirth V. Werner W. Paulissen D. (1992). *Zeigerwerte von Pflanzen in Mitteleuropa.* Göttingen: Erich Goltze KG.

[B15] Ewald J. Hennekens S. Conrad S. Wohlgemuth T. Jansen F. Jenssen M. (2013). Spatial and temporal patterns of Ellenberg nutrient values in forests of Germany and adjacent regions-a survey based on phytosociological databases. *Tuexenia* 33 93–109.

[B16] Federal Office for the Environment (2001). *Biogeographische Regionen der Schweiz.* Available online at: https://opendata.swiss/de/dataset/biogeographische-regionen-der-schweiz-ch) (accessed January, 2016).

[B17] Federal Office of Meteorology and Climatology MeteoSwiss (2018). *Grid Data for Climate Normals.* Zurich: Federal Office of Meteorology and Climatology MeteoSwiss.

[B18] Feng M. Tripathi B. M. Shi Y. Adams J. M. Zhu Y.-G. Chu H. (2019). Interpreting distance-decay pattern of soil bacteria via quantifying the assembly processes at multiple spatial scales. *Microbiologyopen* 8:e00851. 10.1002/mbo3.851 31074596 PMC6741136

[B19] Fierer N. Jackson R. B. (2006). The diversity and biogeography of soil bacterial communities. *Proc. Natl. Acad. Sci. U.S.A.* 103 626–631. 10.1073/pnas.0507535103 16407148 PMC1334650

[B20] Fierer N. McCain C. M. Meir P. Zimmermann M. Rapp J. M. Silman M. R. (2011). Microbes do not follow the elevational diversity patterns of plants and animals. *Ecology* 92 797–804. 10.1890/10-1170.1 21661542

[B21] Frei C. (2014). Interpolation of temperature in a mountainous region using nonlinear profiles and non-Euclidean distances. *Int. J. Climatol.* 34 1585–1605. 10.1002/joc.3786

[B22] Frey B. Rime T. Phillips M. Stierli B. Hajdas I. Widmer F. (2016). Microbial diversity in European alpine permafrost and active layers. *FEMS Microbiol. Ecol.* 92:fiw018. 10.1093/femsec/fiw018 26832204

[B23] Grytnes J.-A. McCain C. M. (2013). “Elevational trends in biodiversity,” in *Encyclopedia of Biodiversity*, 2nd Edn. ed. Levin S. A. (Cambridge, MA: Academic Press), 149–154.

[B24] Hanson C. A. Fuhrman J. A. Horner-Devine M. C. Martiny J. B. H. (2012). Beyond biogeographic patterns: processes shaping the microbial landscape. *Nat. Rev. Microbiol.* 10 497–506. 10.1038/nrmicro2795 22580365

[B25] Harris D. Horwáth W. R. van Kessel C. (2001). Acid fumigation of soils to remove carbonates prior to total organic carbon or CARBON-13 isotopic analysis. *Soil Sci. Soc. Am. J.* 65 1853–1856. 10.2136/sssaj2001.1853

[B26] Hartmann M. Frey B. Kolliker R. Widmer F. (2005). Semi-automated genetic analyses of soil microbial communities: comparison of T-RFLP and RISA based on descriptive and discriminative statistical approaches. *J. Microbiol. Methods* 61 349–360. 10.1016/j.mimet.2004.12.011 15767011

[B27] Hartmann M. Frey B. Mayer J. Mader P. Widmer F. (2015). Distinct soil microbial diversity under long-term organic and conventional farming. *ISME J.* 9 1177–1194. 10.1038/ismej.2014.210 25350160 PMC4409162

[B28] Hervé M. (2018). *RVAideMemoire: Testing and Plotting Procedures for Biostatistics. R Package Version 0.9-69-3.*

[B29] Hintermann U. Weber D. Zangger A. (2000). Biodiversity monitoring in Switzerland. *Landschaftspflege und Naturschutz* 62 42–58.

[B30] International Organization for Standardization (2009). *Soil Quality - Determination of Particle Size Distribution in Mineral Soil Material - Method by Sieving and Sedimentation* (Standard No 11277:2009). Geneva: International Organization for Standardization.

[B31] Isotta F. A. Frei C. Weilguni V. Perčec Tadić M. Lassègues P. Rudolf B. (2014). The climate of daily precipitation in the Alps: development and analysis of a high-resolution grid dataset from pan-Alpine rain-gauge data. *Int. J. Climatol.* 34 1657–1675. 10.1002/joc.3794

[B32] Jaffuel G. Blanco-Pérez R. Hug A.-S. Chiriboga X. Meuli R. G. Mascher F. (2018). The evaluation of entomopathogenic nematode soil food web assemblages across Switzerland reveals major differences among agricultural, grassland and forest ecosystems. *Agric. Ecosyst. Environ.* 262 48–57. 10.1016/j.agee.2018.04.008

[B33] Joimel S. Cortet J. Jolivet C. C. Saby N. P. A. Chenot E. D. Branchu P. (2016). Physico-chemical characteristics of topsoil for contrasted forest, agricultural, urban and industrial land uses in France. *Sci. Total Environ.* 545–546 40–47. 10.1016/j.scitotenv.2015.12.035 26745291

[B34] Karimi B. Terrat S. Dequiedt S. Saby N. Horrigue W. Lelièvre M. (2018). Biogeography of soil bacteria and archaea across France. *Sci. Adv.* 4:eaat1808. 10.1126/sciadv.aat1808 29978046 PMC6031370

[B35] Karimi B. Villerd J. Dequiedt S. Terrat S. Chemidlin-Prévost Bouré N. Djemiel C. (2020). Biogeography of soil microbial habitats across France. *Glob. Ecol. Biogeogr.* 29 1399–1411. 10.1111/geb.13118

[B36] Kielak A. M. Barreto C. C. Kowalchuk G. A. van Veen J. A. Kuramae E. E. (2016). The ecology of *Acidobacteria*: moving beyond genes and genomes. *Front. Microbiol.* 7:744. 10.3389/fmicb.2016.00744 27303369 PMC4885859

[B37] Kindt R. Coe R. (2005). *Tree Diversity Analysis. A Manual and Software for Common Statistical Methods for Ecological and Biodiversity Studies.* Nairobi: World Agroforestry Centre (ICRAF).

[B38] Landolt E. (2010). *Flora Indicativa. Ökologische Zeigerwerte und Biologische Kennzeichen zur Flora der Schweiz und der Alpen.* Bern: Haupt.

[B39] Lauber C. L. Hamady M. Knight R. Fierer N. (2009). Pyrosequencing-based assessment of soil pH as a predictor of soil bacterial community structure at the continental scale. *Appl. Environ. Microbiol.* 75 5111–5120. 10.1128/aem.00335-09 19502440 PMC2725504

[B40] Lauber C. L. Strickland M. S. Bradford M. A. Fierer N. (2008). The influence of soil properties on the structure of bacterial and fungal communities across land-use types. *Soil Biol. Biochem.* 40 2407–2415. 10.1016/j.soilbio.2008.05.021

[B41] Lipson D. A. Raab T. K. Parker M. Kelley S. T. Brislawn C. J. Jansson J. (2015). Changes in microbial communities along redox gradients in polygonized Arctic wet tundra soils. *Environ. Microbiol. Rep.* 7 649–657. 26034016 10.1111/1758-2229.12301

[B42] Mayerhofer J. Eckard S. Hartmann M. Grabenweger G. Widmer F. Leuchtmann A. (2017). Assessing effects of the entomopathogenic fungus *Metarhizium brunneum* on soil microbial communities in *Agriotes* spp. biological pest control. *FEMS Microbiol. Ecol.* 93:fix117. 10.1093/femsec/fix117 PMC5812499 28961941

[B43] Naether A. Foesel B. U. Naegele V. Wüst P. K. Weinert J. Bonkowski M. (2012). Environmental factors affect acidobacterial communities below the subgroup level in grassland and forest soils. *Appl. Environ. Microbiol.* 78 7398–7406. 10.1128/aem.01325-12 22885760 PMC3457104

[B44] Navarrete A. A. Kuramae E. E. de Hollander M. Pijl A. S. van Veen J. A. Tsai S. M. (2013). Acidobacterial community responses to agricultural management of soybean in Amazon forest soils. *FEMS Microbiol. Ecol.* 83 607–621. 10.1111/1574-6941.12018 23013447

[B45] O’Callaghan M. Gerard E. M. Carter P. E. Lardner R. Sarathchandra U. Burch G. (2010). Effect of the nitrification inhibitor dicyandiamide (DCD) on microbial communities in a pasture soil amended with bovine urine. *Soil Biol. Biochem.* 42 1425–1436. 10.1016/j.soilbio.2010.05.003

[B46] Oksanen J. Blanchet G. F. Friendly M. Kindt R. Legendre P. McGlinn D. (2018). *Vegan: Community Ecology Package. R Package Version 2.4-6.*

[B47] Pascual J. García-López M. González I. Genilloud O. (2017). *Luteolibacter gellanilyticus* sp. nov., a gellan-gum-degrading bacterium of the phylum Verrucomicrobia isolated from miniaturized diffusion chambers. *Int. J. Syst. Evol. Microbiol.* 67 3951–3959. 10.1099/ijsem.0.002227 28905697

[B48] Peay K. G. von Sperber C. Cardarelli E. Toju H. Francis C. A. Chadwick O. A. (2017). Convergence and contrast in the community structure of bacteria, fungi and archaea along a tropical elevation–climate gradient. *FEMS Microbiol. Ecol.* 93:fix045. 10.1093/femsec/fix045 28402397

[B49] Pfister R. D. Arno S. F. (1980). Classifying forest habitat types based on potential climax vegetation. *For. Sci.* 26 52–70. 10.1093/forestscience/26.1.52

[B50] Prober S. M. Leff J. W. Bates S. T. Borer E. T. Firn J. Harpole W. S. (2015). Plant diversity predicts beta but not alpha diversity of soil microbes across grasslands worldwide. *Ecol. Lett.* 18 85–95. 10.1111/ele.12381 25430889

[B51] Prosser J. I. Head I. M. Stein L. Y. (2014). “The family Nitrosomonadaceae,” in *The Prokaryotes: Alphaproteobacteria and Betaproteobacteria*, eds Rosenberg E. DeLong E. F. Lory S. Stackebrandt E. Thompson F. (Berlin: Springer Berlin Heidelberg), 901–918.

[B52] Quast C. Pruesse E. Yilmaz P. Gerken J. Schweer T. Yarza P. (2013). The SILVA ribosomal RNA gene database project: improved data processing and web-based tools. *Nucleic Acids Res.* 41 D590–D596. 10.1093/nar/gks1219 23193283 PMC3531112

[B53] Ranjard L. Dequiedt S. Chemidlin Prévost-Bouré N. Thioulouse J. Saby N. P. A. Lelievre M. (2013). Turnover of soil bacterial diversity driven by wide-scale environmental heterogeneity. *Nat. Commun.* 4:1434. 10.1038/ncomms2431 23385579

[B54] R-Core-Team (2016). *R: A Language and Environment for Statistical Computing.* Vienna: R Foundation for Statistical Computing.

[B55] Roth T. Kohli L. Rihm B. Amrhein V. Achermann B. (2015). Nitrogen deposition and multi-dimensional plant diversity at the landscape scale. *R. Soc. Open Sci.* 2:150017. 10.1098/rsos.150017 26064640 PMC4448879

[B56] RStudio-Team (2015). *RStudio: Integrated Development for R.* Boston, MA: RStudio, Inc.

[B57] Schloss P. D. Westcott S. L. Ryabin T. Hall J. R. Hartmann M. Hollister E. B. (2009). Introducing mothur: open-source, platform-independent, community-supported software for describing and comparing microbial communities. *Appl. Environ. Microbiol.* 75 7537–7541 10.1128/aem.01541-09 19801464 PMC2786419

[B58] Shannon P. Markiel A. Ozier O. Baliga N. S. Wang J. T. Ramage D. (2003). Cytoscape: a software environment for integrated models of biomolecular interaction networks. *Genome Res.* 13 2498–2504. 10.1101/gr.1239303 14597658 PMC403769

[B59] Singh D. Lee-Cruz L. Kim W.-S. Kerfahi D. Chun J.-H. Adams J. M. (2014). Strong elevational trends in soil bacterial community composition on Mt. Halla, South Korea. *Soil Biol. Biochem.* 68 140–149. 10.1016/j.soilbio.2013.09.027

[B60] Szoboszlay M. Dohrmann A. B. Poeplau C. Don A. Tebbe C. C. (2017). Impact of land-use change and soil organic carbon quality on microbial diversity in soils across Europe. *FEMS Microbiol. Ecol.* 93:fix146. 10.1093/femsec/fix146 29087486

[B61] Terrat S. Horrigue W. Dequietd S. Saby N. P. A. Lelièvre M. Nowak V. (2017). Mapping and predictive variations of soil bacterial richness across France. *PLoS One* 12:e0186766. 10.1371/journal.pone.0186766 29059218 PMC5653302

[B62] Tripathi B. M. Kim M. Singh D. Lee-Cruz L. Lai-Hoe A. Ainuddin A. N. (2012). Tropical soil bacterial communities in Malaysia: pH dominates in the equatorial tropics too. *Microb. Ecol.* 64 474–484. 10.1007/s00248-012-0028-8 22395784

[B63] Väre H. Lampinen R. Humphries C. Williams P. (2003). “Taxonomic diversity of vascular plants in the European alpine areas,” in *Alpine Biodiversity in Europe*, eds Nagy L. Grabherr G. Körner C. Thompson D. B. A. (Berlin: Springer), 133–147.

[B64] Wang J.-T. Cao P. Hu H.-W. Li J. Han L.-L. Zhang L.-M. (2015). Altitudinal distribution patterns of soil bacterial and archaeal communities along Mt. Shegyla on the Tibetan Plateau. *Microb. Ecol.* 69 135–145. 10.1007/s00248-014-0465-7 25074792

[B65] Wardle D. A. (2006). The influence of biotic interactions on soil biodiversity. *Ecol. Lett.* 9 870–886. 10.1111/j.1461-0248.2006.00931.x 16796577

[B66] Weber D. Hintermann U. Zangger A. (2004). Scale and trends in species richness: considerations for monitoring biological diversity for political purposes. *Glob. Ecol. Biogeogr.* 13 97–104. 10.1111/j.1466-882X.2004.00078.x

[B67] Yashiro E. Pinto-Figueroa E. Buri A. Spangenberg J. E. Adatte T. Niculita-Hirzel H. (2016). Local environmental factors drive divergent grassland soil bacterial communities in the Western Swiss Alps. *Appl. Environ. Microbiol.* 82 6303–6316. 10.1128/aem.01170-16 27542929 PMC5066347

[B68] Zhang Y. Cong J. Lu H. Li G. Qu Y. Su X. (2014). Community structure and elevational diversity patterns of soil Acidobacteria. *J. Environ. Sci.* 26 1717–1724. 10.1016/j.jes.2014.06.012 25108728

