# Supplementary material for: Environmental and Anthropogenic Factors Shape Major Bacterial Community Types Across the Complex Mountain Landscape of Switzerland
Source: Front Microbiol. 2021 Mar 11;12:581430. doi: 10.3389/fmicb.2021.581430 (PMC7990788; doi:10.3389/fmicb.2021.581430)
Supplement: Supplementary file 3 [file Table_1.PDF]

Supplementary Table 1 1
Supplementary Table 2 2
Supplementary Table 3 3
Supplementary Table 4 4
Supplementary Table 5 5
Supplementary Table 6 6
Supplementary Table 7 7
Supplementary Figure 1 8
Supplementary Figure 2 9
Supplementary Figure 3 10
Supplementary Figure 4 11
Supplementary Figure 5 12
Supplementary Figure 6 13
Supplementary File 1 14

Supplementary Table 1: Number of sites included in the reduced (160 sites) and the complete set of sites (255 sites) for each biogeographic region and land-use type.

Level of factor	Factor	Number / percentage of sites per group in reduced set	Number of sites per group in the complete set	Difference of percentages of sites per group between reduced and complete set
Northern Alps	Biogeography	49 / 19 %	74 / 29 %	10 %
Southern Alps	Biogeography	16 / 6 %	28 / 11 %	5 %
Jura Mountains	Biogeography	21 / 8 %	33 / 13 %	5 %
Central Plateau	Biogeography	42 / 16 %	59 / 23 %	7 %
Central Eastern Alps	Biogeography	23 / 9 %	40 / 16 %	7 %
Central Western Alps	Biogeography	9 / 4 %	21 / 8 %	4 %
Alpine grassland	Land-use type	26 / 10 %	47 / 18 %	8 %
Arable land	Land-use type	16 / 6 %	26 / 10 %	4 %
Forest	Land-use type	69 / 27 %	113 / 44 %	17 %
Meadow	Land-use type	42 / 16 %	60 / 24 %	8 %
Settlement	Land-use type	7 / 3 %	9 / 4 %	1 %

Supplementary Table 2: Basic statistics, i.e., mean and standard deviation (SD), of sequences and OTUs per sample and total numbers of sequences and OTUs.

	mean	SD	total
sequences	28,192	10,642	28,474,031
OTUs	3,158	1,084	48,568

Supplementary Table 3: Representation (number and percentage) of sites of each land use type within each biogeographic region.

land use type	Northern Alps	Southern Alps	Jura	Central Plateau	Eastern Central Alps	Western Central Alps
alpine grassland	11 / 15%	4 / 14%	0 / 0%	0 / 0%	24 / 60 %	8 / 38%
arable land	2 / 3%	0 / 0%	6 / 18%	17 / 29%	0 / 0%	1 / 5%
forest	34 / 46%	20 / 71%	16 / 48%	21 / 36%	11 / 28%	11 / 52%
meadow	25 / 34%	3 / 11%	10 / 30%	17 / 29%	4 / 10%	1 / 5%
settlement	2 / 3%	1 / 4%	1 / 3%	4 / 7%	1 / 3%	0 / 0%

Supplementary Table 4: Comparisons of group averages of bacterial community structures among land-use types and biogeographic regions using pairwise PERMANOVA based on BC dissimilarities of site means. Benjamini-Hochberg adjustment was used for p-value correction. Overall tests revealed significant differences of group average among of land-use types (R^2 = 13.6%, p-value = 0.0001) and biogeographic regions (R^2 = 13.0%, p-value = 0.0001).

factor	pairwise comparison	adjusted p-value
land-use type	alpine grassland & arable land	0.0002
	alpine grassland & forest	0.0002
	alpine grassland & meadow	0.0002
	alpine grassland & settlement	0.0002
	arable land & forest	0.0002
	arable land & meadow	0.0008
	arable land & settlement	0.0099
	forest & meadow	0.0002
	forest & settlement	0.0003
	meadow & settlement	0.0072
biogeographic region	Northern Alps & Southern Alps	0.0002
	Northern Alps & Jura	0.0015
	Northern Alps & Central Plateau	0.0008
	Northern Alps & Central Eastern Alps	0.0003
	Northern Alps & Central Western Alps	0.0012
	Southern Alps & Jura	0.0002
	Southern Alps & Central Plateau	0.0002
	Southern Alps & Central Eastern Alps	0.0002
	Southern Alps & Central Western Alps	0.0015
	Jura & Central Plateau	0.0181
	Jura & Central Eastern Alps	0.0002
	Jura & Central Western Alps	0.0002
	Central Plateau & Central Eastern Alps	0.0002
	Central Plateau & Central Western Alps	0.0003
	Central Eastern Alps & Central Western Alps	0.4684[a]

[a] p-value > 0.05

NA not assessed, because overall test was not significant (p > 0.05)

Supplementary Table 5: Effect of each environmental factor individually (marginal tests) on bacterial community structures using distance based linear modelling based on BC dissimilarities of site means. The analyses were based on the reduced dataset of 160 sites. Environmental factors were ordered by decreasing amount of explained variance.

environmental factor	SS(trace)	Pseudo-F	BH* adjusted p-value	explained variance [%]
pH	11.3	65.6	0.0002	29.3
plant indicator for pH	7.1	36.0	0.0002	18.6
plant indicator for nutrients	3.9	18.0	0.0002	10.2
SD of inter-annual precipitation[ab]	2.7	11.7	0.0002	6.9
elevation	2.3	10.2	0.0002	6.1
mean annual temperature[a]	2.1	9.3	0.0002	5.5
clay	2.0	8.7	0.0002	5.2
SD of inter-annual solar radiation[ab]	2.0	8.7	0.0002	5.2
bulk density	2.0	8.6	0.0002	5.2
mean annual precipitation[ac]	1.8	7.7	0.0002	4.7
Slope	1.5	6.5	0.0004	4.0
water content	1.3	5.7	0.0004	3.5
carbon-nitrogen ratio	1.2	5.1	0.0007	3.1
sand	1.2	4.9	0.0005	3.0
organic carbon	0.8	3.5	0.0060	2.2
mean solar radiation[ac]	0.8	3.2	0.0093	2.0
SD inter-annual temperature[ae]	0.7	3.1	0.0093	1.9
plant indicator for moisture	0.7	3.0	0.0109	1.9
plant indicator for variability of moisture	0.6	2.4	0.0350	1.5
total nitrogen	0.5	1.9	0.0673	1.2
silt	0.4	1.9	0.0697	1.2
DNA content	0.3	1.1	0.2814	0.7
exposition	0.2	1.0	0.3615	0.6

* Benjamini & Hochberg

SD standard deviation

[a] based on values between 1981-2010

[b] mean of standard deviation of monthly sums

[c] mean of yearly sums

[e] mean of monthly standard deviation

Supplementary Table 6: Selection of a distance based linear model of bacterial community structures based on BC dissimilarities of mean relative sequence abundances for each site. The analysis was performed with the complete dataset of environmental factors which included 160 sites. Factors were selected in a step-wise manner using AICc selection criterion and p-values were obtained from 9999 permutations.

environmental factor	AICc	SS(trace)	pseudo-F	p-value	explained variation [%]	cumul. Explained variation [%]	residual df
pH	-279.9	11.3	65.6	0.0001	29.3	29.3	158
plant indicator for nutrients	-291.9	2.3	14.5	0.0001	6.0	35.3	157
elevation	-297.0	1.1	7.3	0.0001	2.9	38.2	156
clay	-300.5	0.8	5.5	0.0001	2.1	40.3	155
bulk density	-302.9	0.6	4.5	0.0001	1.7	42.0	154
soil water content	-305.3	0.6	4.5	0.0001	1.6	43.6	153
plant indicator for pH	-307.1	0.5	3.8	0.0001	1.4	45.0	152
plant indicator for moisture	-308.0	0.4	3.1	0.0005	1.1	46.1	151
plant indicator for variability of moisture	-308.5	0.3	2.6	0.0014	0.9	47.0	150
SD of inter-annual precipitation[a]	-308.9	0.3	2.5	0.0013	0.9	47.9	149

SD standard deviation

[a] mean of standard deviation of monthly sums between 1981 and 2010

Supplementary Table 7: Difference of means of environmental factors among six bacterial community derived clusters (BCT I to VI; ANOVA). Benjamini-Hochberg adjustment was used for p-values correction.

	number of sites	F-statistic	adjusted p-value	explained variance [%]
pH	236	241.5	4.96E-88	83.7
plant indicator for pH	255	76.8	2.10E-47	59.9
plant indicator for nutrients	255	48.1	8.22E-34	48.1
elevation	255	32.8	6.50E-25	38.5
mean annual temperature[a]	255	27.4	2.16E-21	34.2
clay	194	14.2	2.31E-11	25.5
bulk density	238	16.3	3.24E-13	24.4
SD of inter-annual solar radiation[ab]	255	15.3	1.46E-12	21.9
carbon-nitrogen ratio	225	13.2	7.17E-11	21.4
slope	255	14.3	6.98E-12	20.8
organic carbon	225	11.0	3.42E-09	18.3
SD of inter-annual precipitation[ab]	255	11.9	5.60E-10	17.6
sand	194	7.0	8.61E-06	13.5
mean of annual precipitation[ac]	255	5.6	1.08E-04	8.3
mean solar radiation[ac]	255	5.0	3.29E-04	7.3
soil water content	238	3.9	2.91E-03	5.8
total nitrogen	231	3.4	6.98E-03	5.0
plant indicator for variability of moisture	255	3.6	4.82E-03	4.9
plant indicator for moisture	255	3.1	1.26E-02	3.9
DNA content	238	2.9	1.62E-02	3.9
silt	194	2.4	4.08E-02	3.5
SD inter-annual temperature[ad]	255	2.8	2.02E-02	3.4
exposition	224	1.0	4.24E-01	0.0

SD standard deviation

[a] based on values between 1981-2010

[b] mean of standard deviation of monthly sums

[c] mean of yearly sums

[d] mean of monthly standard deviation

Supplementary Figure 1: Map of 160 sites (blue triangles) for which all environmental data were available (reduced set) and additionally 95 sites (red circles) for which only subsets of environmental data were available.

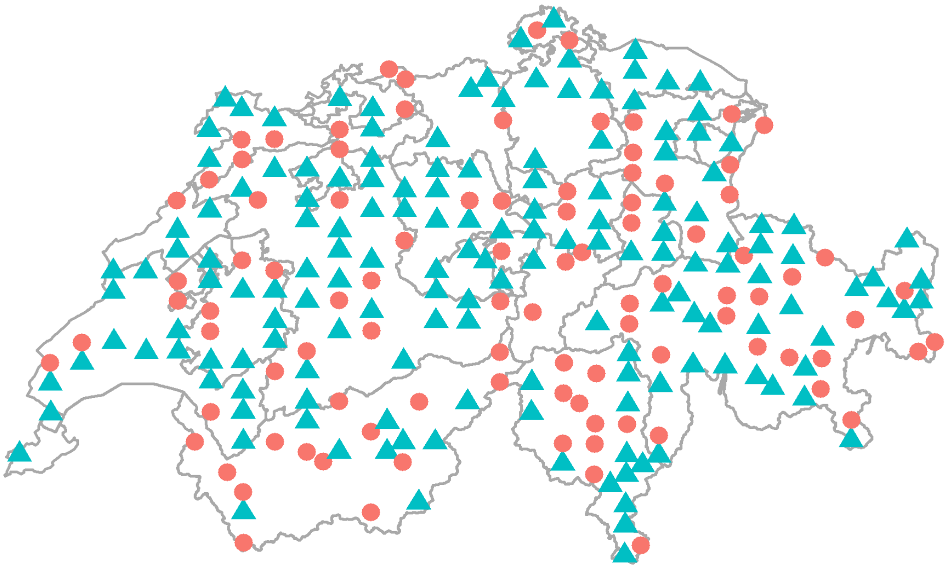

Supplementary Figure 2: Cluster of bacterial community types. Tree displaying sites based on their similarity of bacterial community structures (A; Bray Curtis dissimilarity). Clustering was performed using hierarchical agglomeration and the Ward's minimum variance method (Ward D2). Schematic display of bacterial community types (BCTs) A, B, A1, B1, I, II, III, IV, V, VI based on clustering (B).

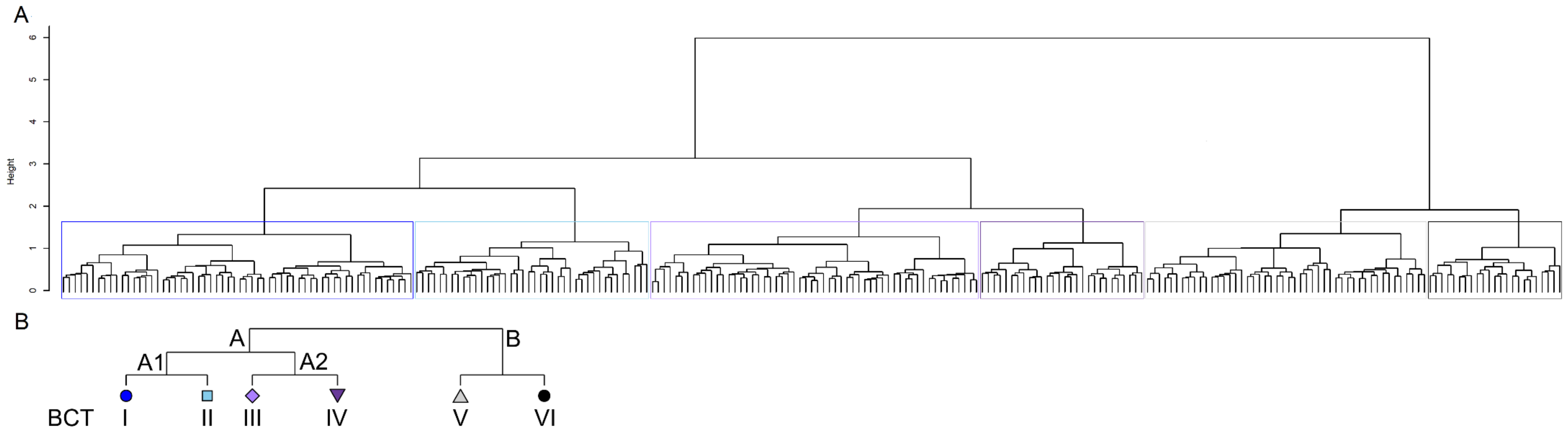

Supplementary Figure 3: Bacterial community structures among biogeographic regions in panel A, including Northern Alps (NA), Jura Mountains (JM), Central Plateau (CP), Eastern Central Alps (EA) and Western Central Alps (WA) and Southern Alps (SA), and land-use types in panel B, including alpine grassland (AG), arable land (AL), forest (FO), meadow (ME) and settlement (SE). Total allocation success of sites to biogeographic regions and land-use types was 66.7% and 85.5% using the leave-one-out procedure of the canonical analysis of principal coordinates (CAP). Reclassification success per group level is presented within the figure legend. Points and stars show correctly and incorrectly reclassified samples, respectively. Unconstrained analyses (PERMANOVA) showed that 13.0 and 13.6% of the variance in bacterial communities were explained by the factors 'biogeographic region' and 'land-use type'.

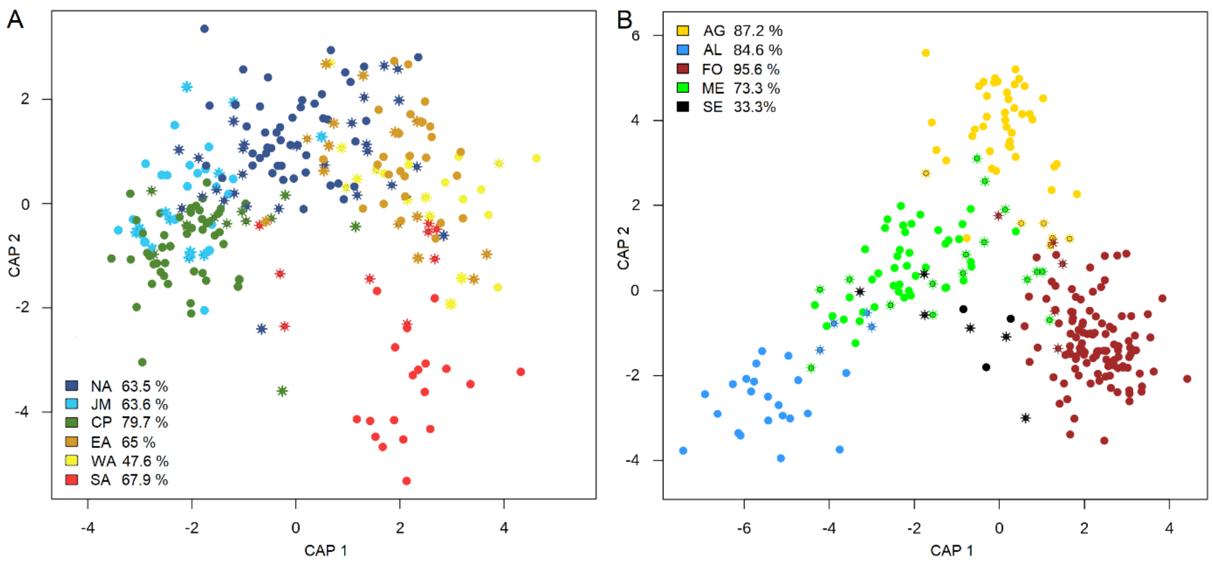

Supplementary Figure 4: Values of environmental factors among sites at which different bacterial community types (BCTs) occurred, i.e., BCT I (dark blue; n=60), BCT II (light blue; n=40), BCT III (light purple; n=56) to BCT IV (dark purple; n=28), BCT V (grey; n=48) and BCT VI (black; n=23). Plots were ordered by decreasing effect size (ANOVA, pairwise Tukey test, BH adjusted p-value < 0.05) and included pH, plant indicator for pH (R), plant indicator for nutrients (N), elevation, mean annual temperature, clay content, bulk density, standard deviation of inter-annual solar radiation, carbon to nitrogen ratio (CN), slope, organic carbon, standard deviation of inter-annual precipitation, sand content, mean annual precipitation, mean annual solar radiation, soil water content, total nitrogen, plant indicator for moisture(F), plant indicator for variability in moisture (W) and soil DNA content.

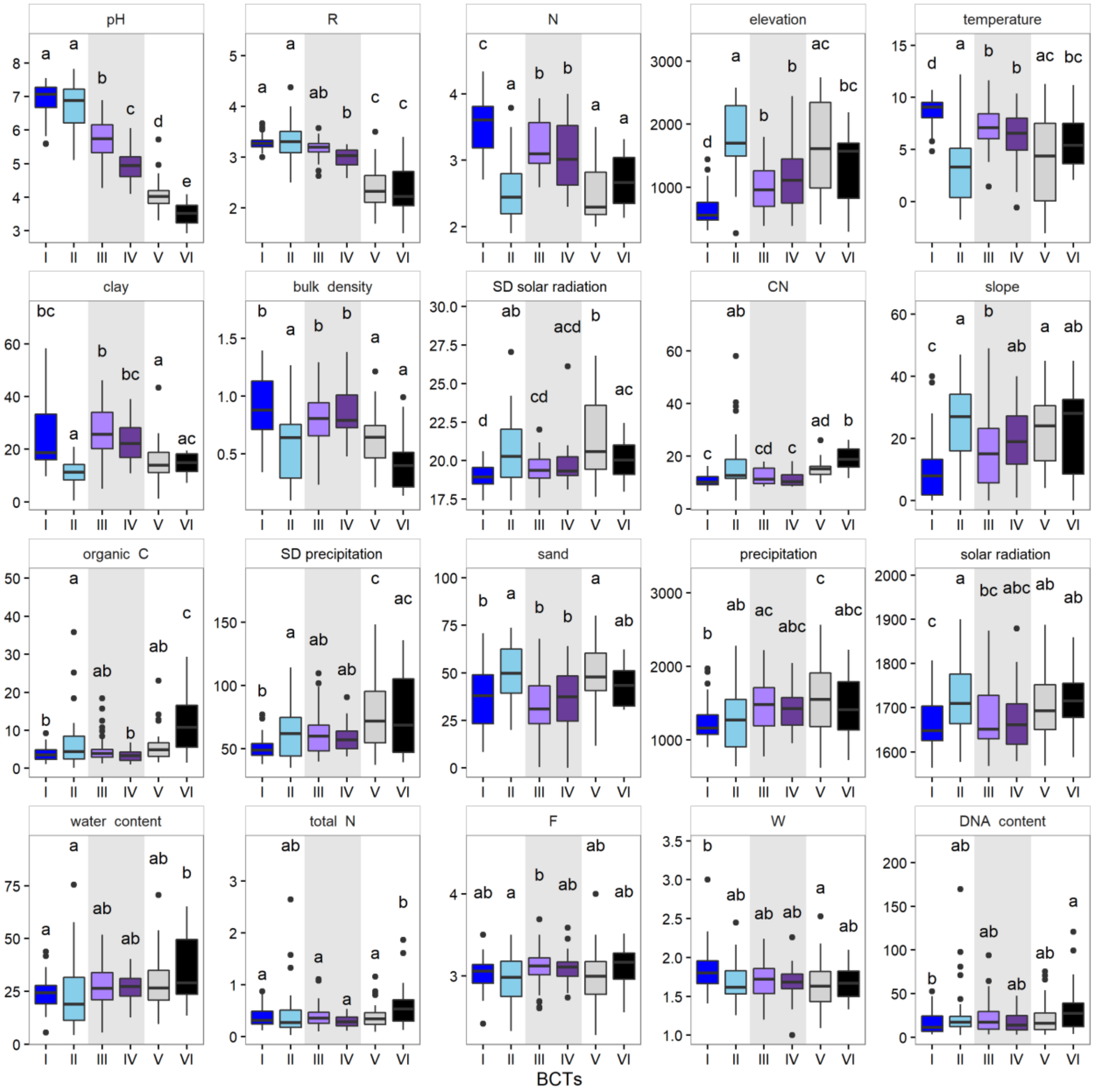

Supplementary Figure 5: Geographic distribution of bacterial community types, i.e., BCT I to VI (A-F) across Switzerland. Biogeographic regions include the Jura Mountains (light blue), the Central Plateau (green), the Northern Alps (dark blue), the Western Central Alps (yellow), the Eastern Central Alps (orange) and the Southern Alps (red).

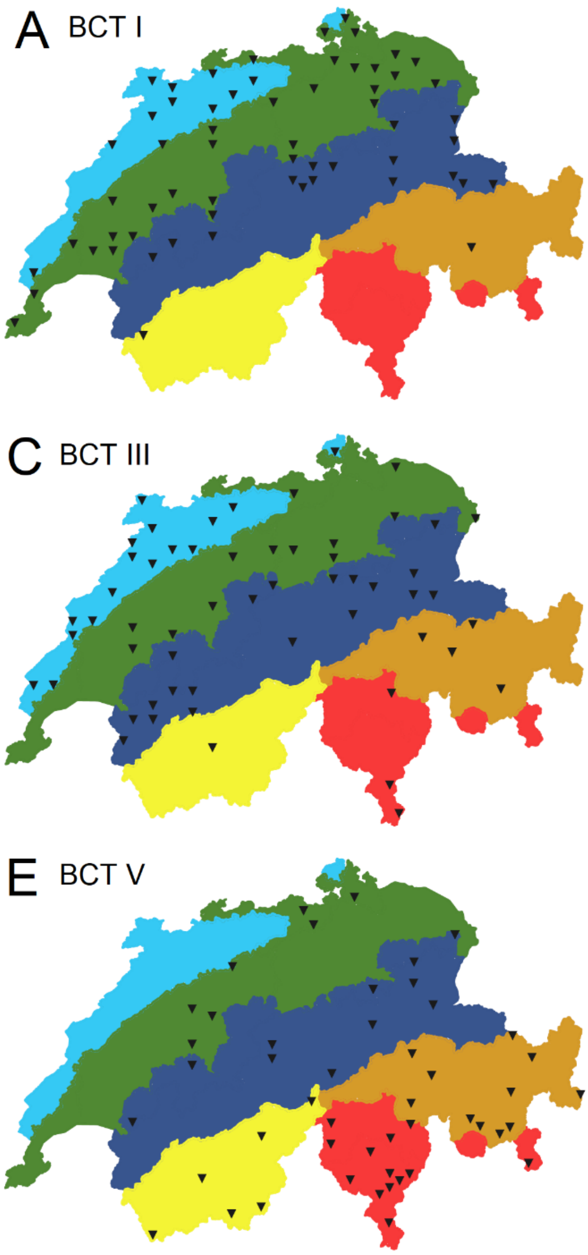

Supplementary Figure 6: OTUs associated to different environmental factors such as the indicator value for soil pH (R; in total 1,406 OTUs), elevation (EL; in total 47 OTUs), bulk density (BD; in total 70 OTUs), soil water content (SWC; in total 33 OTUs) and standard deviation of inter-annual precipitation (sdPR; in total 148 OTUs). Environmental factors are represented by grey octagons and levels of each factor by grey squares. Levels of continuous factors include open intervals on the left side and closed intervals on the right side. OTUs associated to the same level or levels are represented by white circles. Numbers in the circles represent the number of all OTUs, which were significantly associated to the same level or levels of a factor. The diameters of the white circles represent the relative abundance of the groups of associated OTUs.

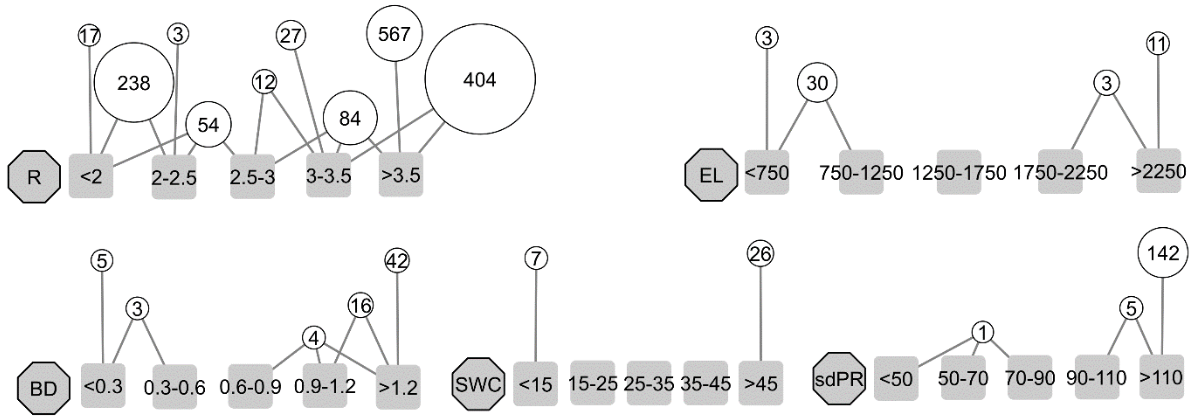

Supplementary File 1

```
# Bioinformatic pipeline metabarcoding Illumina

# concatenate sequences of all files
cat *label.R1.fastq > 1.all.R1.fastq && cat *label.R2.fastq > 1.all.R2.fastq

# exclude PhiX sequences
usearch -filter_phix 1.all.R1.fastq -reverse 1.all.R2.fastq -output 2.all.nophix.R1.fastq -
output2 2.all.nophix.R2.fastq -alnout phiX_hits.txt

# merge paired-end reads
usearch -fastq_mergepairs 2.all.nophix.R1.fastq -reverse 2.all.nophix.R2.fastq -
fastq_minovlen 50 -fastq_minmergelen 300 -fastq_maxdiffs 250 -fastq_maxdiffpct 15 -
fastq_allowmergestagger -fastqout 3.merge.fastq -log 3.merge.log -report 3.merge.report.txt

# strip PCR primers
cutadapt -g ^CCTAYGGGDBGCWSCAG -e 0.06 -f fastq --trimmed-only -o 4.cutprim1.fastq
3.merge.fastq
cutadapt -g ATTAGADACCCBNGTAGTCC$ -e 0.06 -f fastq --trimmed-only -o
4.cutprim2.fastq 4.cutprim1.fastq
cutadapt -g ^CCTAYGGGDBGCWSCAG -a ATTAGADACCCBNGTAGTCC$ -n 10 -e
0.06 -f fastq -o 4.cutprim.fastq 4.cutprim2.fastq

# quality filter based on maximum expected error
usearch -fastq_filter 4.cutprim.fastq -fastqout 5.maxee1.fastq -fastq_maxee 1

# dereplicate sequences
usearch -fastx_uniques 5.maxee1.fastq -fastaout 6.all.derep.fasta -sizeout -minuniquesize 2

#cluster into OTUs and remove chimera
usearch -cluster_otus 6.all.derep.fasta -otu_radius_pct 3 -otus 6.OTU.derep.fasta -uparseout
6.OTU.derep.up -relabel OTUb_

#target verification
metaxa2_x -i 6.OTU.derep.fasta -o 7.OTU.derep.fasta.metaxa --complement F --cpu 16

#map all sequences to seed and make otu table
usearch -usearch_global 5.maxee1.fastq -db 7.OTU.derep.fasta.metaxa.extraction.clean.fasta -
strand plus -id 0.97 -maxaccepts 0 -top_hit_only -maxrejects 0 -uc 8.OTU.clean.map.uc -
matched 8.OTU.clean.map.fasta –otutabout 8.OTU.clean.map

# taxonomic classification SILVA 132
mothur "#classify.seqs(fasta=7.OTU.derep.fasta.metaxa.extraction.clean.fasta,
template=SILVA.fasta, taxonomy=SILVA.tax, cutoff=80)"
```
